# Resolving conformational polymorphism in disulfide-rich peptide drugs

**DOI:** 10.3389/fchem.2026.1913741

**Published:** 2026-08-17

**Authors:** Shaozhou Zhu, Yifeng Ge, Haiwei Huang, Mingzhe Xu

**Affiliations:** 1 Institute for Chemical Drug Control, National Institute for Food and Drug Control, Beijing, China; 2 College of New Materials and Chemical Engineering, Beijing Institute of Petrochemical Technology, Beijing, China

**Keywords:** cyclic ion mobility mass, disulfide-rich peptide, linaclotide, quality control, topological impurity, ziconotide

## Abstract

Disulfide-rich peptide therapeutics often exhibit compact cystine knot-like architectures that endow them with high conformational rigidity, proteolytic stability, and target specificity. Yet their intrinsic polymorphism and structural heterogeneity are difficult to resolve with conventional analytical methods, which lack sufficient power to distinguish subtle structural variants within highly constrained peptide frameworks. Here, cyclic ion mobility-mass spectrometry (cIM-MS), combined with accelerated thermal stress testing, was applied to a manufacturing batch of ziconotide and linaclotide under native and thermally stressed conditions. Pronounced conformational heterogeneity was observed for both drugs even under native conditions. Upon thermal stress, notable degradation and structural reorganization occurred, revealing conformational changes that were not adequately captured by conventional methods. The high-resolution separation achieved by cIM-MS enabled detailed mapping of coexisting conformers and their stress-induced transitions. These results demonstrate that cIM-MS provides a spatially resolved analytical platform for interrogating higher-order structural heterogeneity in disulfide-rich peptide therapeutics, extending the capabilities of conventional mass spectrometry and offering a generalizable approach for structural characterization and quality assessment.

## 1 Introduction

Peptides rich in disulfide bonds represent a prominent class of biologically active macromolecules ubiquitous across diverse domains of life (Góngora-Benítez et al., 2014). Their hallmark cysteine knot (or cystine knot) architecture frequently confers exceptional conformational rigidity and resistance to proteolytic degradation, enabling these molecules to retain functionality in complex and even harsh physiological or environmental conditions (Craik et al., 2001). This structural motif is commonly found in hormone families, toxins, and plant-derived cyclotides, underscoring its profound biological significance and substantial potential for translational applications (Craik et al., 2001; Venkatesan and Roy, 2023).

**FIGURE 1:**
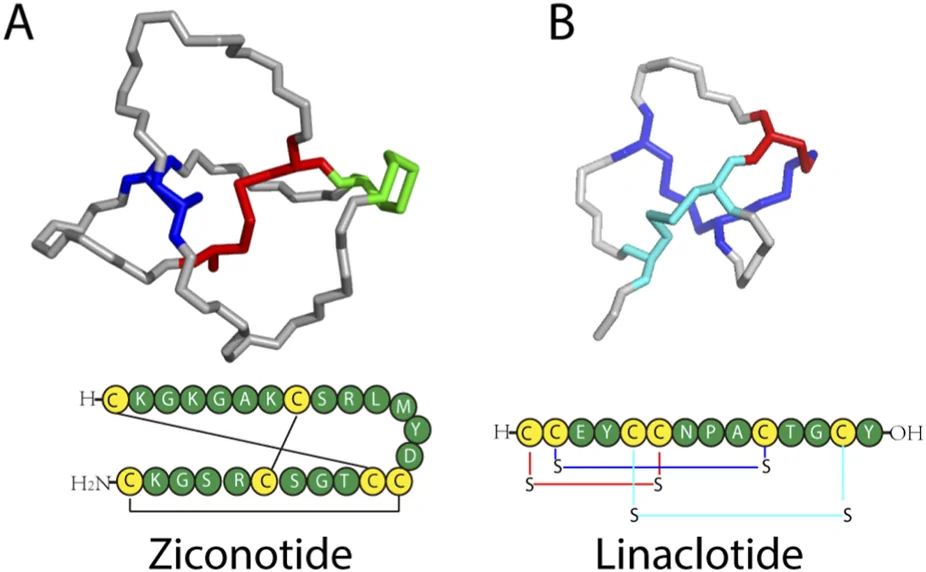
Structures of representative disulfide-rich peptide drugs. **(A)** ziconotide (Generated from PDB:7MIX); **(B)** linaclotide (Generated by Boltz-1).

Representative examples of disulfide-rich peptide drugs include ω-conotoxin MVIIA (ziconotide) and linaclotide (Bassotti et al., 2018; Lin et al., 2024; Love et al., 2014; Schmidtko et al., 2010). Ziconotide, a 25-amino-acid peptide, is an FDA-approved analgesic, while linaclotide, a 14-amino-acid orally active guanylate cyclase-C receptor agonist, is employed therapeutically for gastrointestinal disorders (Lin et al., 2024; Love et al., 2014). Both adopt a compact cysteine knot folding pattern, a feature critical to their druggability. Specifically, ziconotide possesses six cysteine residues that form three disulfide bonds, thereby stabilizing its polypeptide conformation (Figure 1) (Lin et al., 2024; Schmidtko et al., 2010); linaclotide similarly features three disulfide bridges that markedly enhance its molecular stability (Love et al., 2014). Owing to their high target specificity combined with superior resistance to enzymatic degradation, disulfide-rich peptides have emerged as highly attractive lead compounds and chemical probes in drug discovery (Góngora-Benítez et al., 2014).

Nevertheless, the pronounced rigidity and dense disulfide network of these molecules render their folding pathways considerably more intricate, posing significant challenges for structural elucidation and quality control (Cheng et al., 2024; Cheng and Wu, 2025). Despite their enhanced stability, such peptides often exhibit intrinsic conformational polymorphism and topological heterogeneity-phenomena that remain incompletely understood (Banerjee et al., 1996). In theory, peptides bearing three disulfide bonds can generate a combinatorially large number of disulfide connectivity isomers during oxidative folding (Durand et al., 2017; Zheng et al., 2017). In biological systems, however, typically only one “native” conformation possesses full biological activity. Folding processes nonetheless transiently produce multiple intermediates and mispaired isomers, substantially complicating synthesis and analytical characterization (Zheng et al., 2017). Studies have demonstrated, for instance, that incomplete or incorrect disulfide pairing can abolish pharmacological activity entirely (Yao et al., 2022). Even after correct folding, subtle conformational variants may still remain. These topological isomers have the same amino acid sequence and disulfide connectivities, but differ in their three-dimensional topological arrangement. Such cryptic isomeric forms add substantial complexity to the system, and a key knowledge gap remains regarding how disulfide-rich therapeutics are distributed among conformational states under manufacturing and storage conditions (Góngora-Benítez et al., 2014).

Notably, conventional analytical techniques often fail to resolve these subtle conformational distinctions in peptides. Routine liquid chromatography typically cannot differentiate isomers with identical retention time, while nuclear magnetic resonance (NMR) spectroscopy predominantly captures the dominant native conformation, rendering low abundance or transient states difficult to detect (Fujinami et al., 2020; Wu et al., 2000). Although conventional LC-MS is widely used for the characterization of peptide therapeutics, direct evaluation of conformational changes associated with disulfide bond integrity remains challenging (Chaturvedi et al., 2024). Disulfide-linked species often possess identical molecular masses, preventing conventional LC-MS from readily distinguishing conformers or disulfide isomers. Consequently, disulfide characterization generally relies on reduction/non-reduction strategies coupled with alkylation, enzymatic digestion, and peptide mapping (Yen et al., 2002). Such workflows are labor-intensive, require extensive sample preparation, and may introduce disulfide bond scrambling or other preparation-induced artifacts. Moreover, disruption of the native protein structure during sample processing limits the ability to assess conformational changes while preserving near-native structural features. In contrast, ion mobility-mass spectrometry (IM-MS) provides orthogonal separation based on molecular shape, offering novel discriminatory power (Kanu et al., 2008; Lanucara et al., 2014; Paglia et al., 2015; Ruotolo et al., 2008). For example, recent studies employing matrix-assisted laser desorption/ionization-ion mobility-mass spectrometry (MALDI-IM-MS) revealed conformational preferences of cyclic versus linear Gramicidin S in the gas phase, with protonated and sodiated forms favoring β-sheet (or β-hairpin) structures, while protonated cyclic variants tended toward collapsed random coil conformations (Ruotolo et al., 2004). In contrast to conventional IMS, the recently developed cIMS enables ions to undergo multiple passes through a cyclic separation device, thereby effectively extending the ion mobility separation path. This design provides enhanced mobility resolving power, enabling improved discrimination of ions with subtle differences in collision cross section, including closely related conformers that often remain unresolved by conventional IMS. These capabilities make cIMS particularly valuable for probing conformational heterogeneity and establishing high-resolution ion mobility fingerprints of peptide and protein therapeutics. For example, cIMS has been successfully applied to distinguish aspartic acid isomerization from isoaspartic acid formation in model peptides, revealing distinct conformational profiles for each isomer. When combined with straightforward ion mobility data comparison workflows, the approach also enabled precise localization of isomerization sites, highlighting the potential of cIMS for resolving subtle structural variations that are difficult to detect by conventional MS-based methods (Gibson et al., 2022; Giles et al., 2019).

To date, systematic investigations of conformational isomers in approved disulfide-rich peptide therapeutics remain limited. Herein, we focus on ziconotide and linaclotide as model compounds to systematically examine their conformational polymorphism. Our findings confirm the presence of varying proportions of conformational isomers in the corresponding drug substance batches. The superior resolution afforded by cIM-MS enabled precise characterization of conformational heterogeneity in these therapeutics.

In summary, our work demonstrates that cIM-MS constitutes a powerful conformational-resolution analytical platform for quality assessment of complex peptide therapeutics. By revealing conformational heterogeneity undetectable by conventional mass spectrometry, cIM-MS facilitates more comprehensive structural characterization. This approach introduces an additional “conformational dimension”, ensuring detection and monitoring of even trace-level structural variants. More broadly, the analytical framework established herein is readily extensible to other peptides and proteins with complex structures, offering new avenues for correlating folding topology with function and stability, and thereby advancing mechanistic understanding of peptide therapeutics as well as regulatory science in this domain.

## 2 Materials and methods

### 2.1 Materials

Ziconotide (batch no. 282816) and linaclotide (batch no. 1083723) were purchased from MedChemExpress LLC (5 mg per vial). Stlassin (3.4 mg/vial) was kindly provided by Prof. Ming Ma (Peking University, Beijing, China). Chromatography-grade acetonitrile was obtained from Honeywell International Inc., and chromatography-grade trifluoroacetic acid and DL-Dithiothreitol were sourced from Sigma-Aldrich. Ultrapure water (18.2 MΩ·cm at 25 °C, TOC <5 ppb) was prepared using a Milli-Q Gradient system (Merck Millipore, Burlington, MA, United States). The labeled purity of the ziconotide reference material was 99.23%, and that of linaclotide was 99.77%. All peptide reference materials were stored at −20 °C and protected from light.

### 2.2 Instruments

Chromatographic analyses were performed using an UltiMate 3000 HPLC system (Thermo Fisher Scientific). Ion mobility-mass spectrometry measurements were acquired on a Select Series Cyclic IMS instrument (Waters Corporation). Mass measurements were obtained using an XPR206 analytical balance (0.01 mg readability; Mettler Toledo).

### 2.3 Solution preparation

Ziconotide (0.72 mg) and linaclotide (1.11 mg) were accurately weighed and transferred separately into 50 mL volumetric flasks. Each compound was dissolved and diluted to volume with ultrapure water and the solutions were mixed thoroughly to yield individual peptide solutions with concentrations of approximately 20 μg/mL. For reference, 0.904 mg of Stlassin was accurately weighed and transferred into a 50 mL volumetric flask, and diluted to volume to prepare a solution with a concentration of approximately 20 μg/mL.

For thermal stability testing, three aliquots (2.0 mL each) of each peptide solution were transferred into centrifuge tubes and incubated in metal heating baths at 0, 50, and 80 °C for 1 h. After incubation, these samples were immediately placed on ice after each time point to quench the treatment and were used as test solutions for HPLC and cIM-MS analyses. For HPLC analysis, one aliquot of the sample was analyzed directly without centrifugation, whereas the other aliquot was centrifuged at 13,000 rpm for 5 min prior to analysis. For disulfide bond reduction, aliquots (2.0 mL each) of peptide solutions were treated with 50 μL of DTT solution (0.5 M) and incubated at 37 °C for 1 h. All experiments were performed in duplicate.

### 2.4 Analysis by HPLC and LC-MS

Heat-treated ziconotide and linaclotide were initially subjected to high-performance liquid chromatography (HPLC) for separation analysis. Chromatographic separation was performed using a Welch Ultimate XB C18 column (150 mm × 4.6 mm, 5 μm particle size). The mobile phase consisted of 0.1% trifluoroacetic acid in water (solvent A) and 0.1% trifluoroacetic acid in acetonitrile (solvent B). Gradient elution was programmed as follows: initial conditions of 90% A and 10% B were held from 0 to 2 min, followed by a linear gradient to 50% A and 50% B from 2 to 15 min, then to 5% A and 95% B from 15 to 25 min, held at 5% A and 95% B from 25 to 30 min, returned to 90% A and 10% B from 30 to 31 min, and equilibrated at 90% A and 10% B from 31 to 35 min. Analyses were conducted at a flow rate of 1 mL/min, with UV detection at 220 nm, column temperature maintained at 30 °C, and an injection volume of 20 μL.

LC-MS analyses were performed on a Waters Acquity UPLC system coupled to a SELECT SERIES Cyclic IMS mass spectrometer (Waters, Milford, MA, United States) equipped with an electrospray ionization (ESI) source. Chromatographic separation was achieved on a ACQUITY UPLC BEH Shield RP C18 column (100 × 2.1 mm, 1.7 μm) using water containing 0.1% (v/v) trifluoroacetic acid (solvent A) and acetonitrile containing 0.1% (v/v) trifluoroacetic acid (solvent B). LC-MS data were acquired in positive-ion mode (ESI^+^) over an *m/z* range of 50–2,000 using an isocratic mobile phase of A:B = 50:50 (v/v). The ESI source was operated under the following conditions: capillary voltage, 3.0 kV; cone voltage, 40 V; source temperature, 150 °C; desolvation temperature, 280 °C; desolvation gas flow, 800 L/h; and reference capillary voltage, 3.0 kV. For MS/MS analysis, the most abundant charge state was selected as the precursor ion and fragmented by collision-induced dissociation (CID).

### 2.5 Analysis by TOF-MS and cIM-MS

TOF-MS analyses were performed in positive electrospray ionization (ESI^+^) mode with a scan range of *m/z* 50–2000. cyclic IM-MS analyses were carried out by direct infusion at a flow rate of 5 μL/min. The critical cyclic IMS parameters were set as follows: racetrack bias, 70 V; array offset, 2 V; array traveling wave velocity, 375 m/s; and array traveling wave height, 4 V. The mobility separation time was adjusted as appropriate. All other instrument parameters are provided in Supplementary Table S1. All experiments were performed in duplicate.

### 2.6 Optimization of operating parameters in cyclic ion mobility mass spectrometry

Using ziconotide as a model analyte, we optimized the key operating parameters of cIM-MS. We focused on the global parameters that exert the strongest influence on ion transmission efficiency and ion mobility separation, including the racetrack bias and the array offset. During optimization, a one-parameter-at-a-time strategy was adopted, with all other instrumental settings held constant. The racetrack bias was varied from 50 to 90 V to optimize ion transmission prior to entry into the cyclic ion mobility cell. The array offset was adjusted within the range of 50–70 V to control ion injection from the array region into the racetrack and to ensure stable ion transmission during subsequent mobility separation. In the cIM-MS workflow, ions were introduced from the array into the racetrack, subjected to multiple rounds of mobility separation under the preset conditions, and then ejected for detection. The number of mobility passes was subsequently optimized within this separation scheme.

### 2.7 cIM-MS mobility separation experiments for ziconotide and linaclotide

cIM-MS mobility separation experiments were performed using ziconotide and linaclotide as model cyclic peptide analytes under optimized instrumental conditions. For ziconotide, mobility separation was conducted using the triply protonated ion ([M+3H]^3+^, m/z 880.079) at drift times of 2, 14, 23, and 55 ms. To assess the effect of charge state on ion mobility behavior, the quadruply protonated ion ([M+4H]^4+^, m/z 660.308) was additionally analyzed under comparable separation conditions. For linaclotide, mobility separation experiments were carried out using the doubly charged sodium-adduct ion ([M+4Na-2H]^2+^ (m/z 807.715). Drift times of 2, 14, 23, 40, and 50 ms were applied to systematically vary the effective ion mobility separation time. For Stlassin, mobility separation experiments were carried out using the quadruply protonated ion ([M+2Na+2H]^4+^ (m/z 870.975). Drift times of 5, 55 and 115 ms were applied to systematically vary the effective ion mobility separation time. For the DTT-reduced samples, mobility separation was performed using the triply protonated ion of ziconotide ([M+3H]^3+^, *m/z* 882.043) and the triply protonated ion of linaclotide ([M+3H]^3+^, *m/z* 810.672).

In all experiments, ions were introduced into the cyclic mobility device and subjected to multiple passes along the cyclic drift path prior to ejection and detection. The number of passes, corresponding to the effective drift time, was controlled by adjusting the separation duration. All measurements were performed under the optimized cIM-MS conditions described above.

### 2.8 Slice-based cIM-MS analysis of mobility-selected ion subpopulations

Slice-based ion mobility selection experiments were performed using the built-in slicing function of cIM-MS to enable targeted analysis of mobility-resolved ion subpopulations.

Following an initial mobility separation (23 ms), arrival time distributions of ziconotide and linaclotide ions were divided into defined temporal regions. The leading and trailing halves of the distributions were selectively isolated using the slice function, corresponding to distinct ion subpopulations within the mobility profile. Each isolated subpopulation was subsequently reinjected into the cyclic drift path and subjected to an additional 23 ms of ion mobility separation under identical instrumental conditions.

All slicing experiments were conducted under the optimized cIM-MS parameters described above. Ion selection windows and subsequent mobility separations were controlled to ensure reproducible isolation and analysis of mobility-resolved ion subpopulations.

### 2.9 Thermal stress and ion mobility fingerprint profiling of peptide drugs

Ziconotide and linaclotide were subjected to thermal stress to assess degradation behavior using ion mobility fingerprint profiling based on cIM-MS. Both peptides were incubated at 50 °C for defined time periods to induce accelerated degradation. Following thermal treatment, each sample was analyzed by cIM-MS using a 50 ms drift separation. Three-dimensional ion mobility mapping was acquired for all detected ions, enabling visualization of ion distributions across the drift time and retention time axes. Mass spectrometric detection was performed to identify newly generated ions corresponding to degradation products, including multi-sodiated species.

All cIM-MS measurements were conducted under the optimized instrumental conditions described above. Ion mobility fingerprint data were collected and processed to generate drift time versus retention time heatmaps and three-dimensional ion distribution profiles, providing a systematic method for evaluating changes in ion abundance and the formation of degradation products under thermal stress.

## 3 Results

### 3.1 HPLC and LC-MS is inadequate for characterizing the conformational heterogeneity of disulfide-rich peptide drugs

**FIGURE 2:**
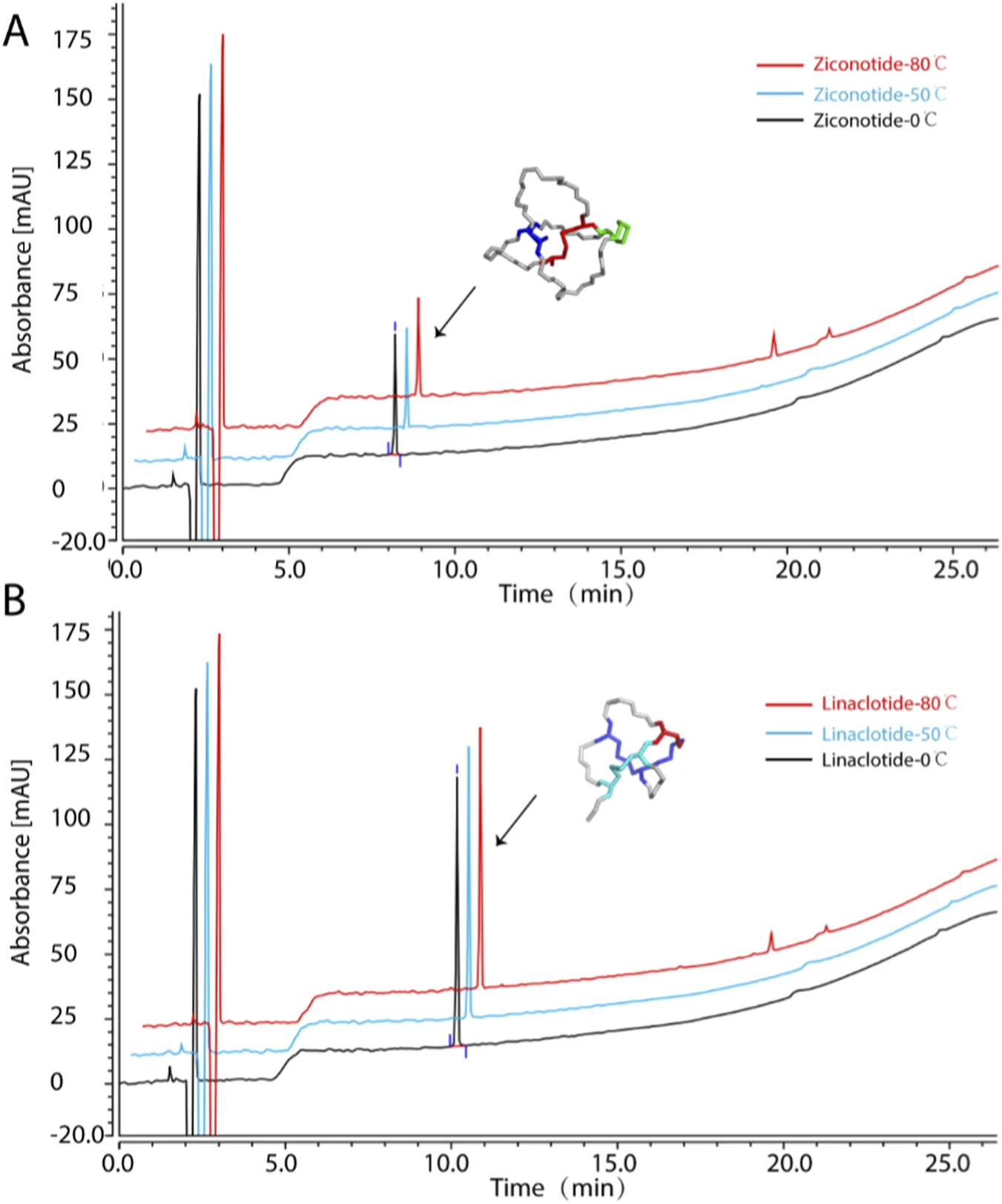
Temperature-dependent C18 HPLC profiles of ziconotide and linaclotide after heat treatment. **(A)** ziconotide; **(B)** linaclotide.

C18 reversed-phase high-performance liquid chromatography (HPLC) was employed to assess the conformational separation and degradation of ziconotide and linaclotide. Samples subjected to different temperature treatments were transferred to HPLC vials and analyzed under a predetermined gradient (Figure 2). As shown in Figure 2 and Supplementary Figure S1, the main peak of ziconotide eluted at approximately 8.6 min and remained essentially unchanged across the three temperatures examined, with no significant retention time shifts. Minor degradation products were observed following incubation at 80 °C, but their abundance was low. Compared with the untreated sample, the main peak area of ziconotide showed no obvious change after incubation at 50 °C for 1 h, whereas it decreased by 7.6% ± 1.9% after treatment at 80 °C for 1 h when the samples were not centrifuged (Supplementary Figure S1A). Considering that peptide or protein therapeutics may undergo heat-induced aggregation and subsequent precipitation during centrifugation, HPLC analysis was also performed on centrifuged samples to assess the potential impact of centrifugation on the chromatographic profiles. when the samples were centrifuged at 13,000 rpm for 5 min, the decrease increased to 17.9% ± 2.2% (Figure 2A), suggesting that ziconotide underwent aggregation during thermal treatment.

Similarly, linaclotide exhibited a main peak at approximately 11.0 min, which remained stable under all three temperature conditions, with only modest degradation detected after 1 h of treatment at 80 °C. Compared with the untreated sample, the main peak area showed no obvious change after incubation at 50 °C for 1 h, whereas it decreased by 3.3% ± 1.5% after treatment at 80 °C for 1 h when the samples were not centrifuged (Supplementary Figure S1B). When the samples were centrifuged at 13,000 rpm for 5 min, the decrease was 5.2% ± 0.6% (Figure 2B), indicating that linaclotide did not undergo pronounced aggregation during thermal treatment, in contrast to ziconotide.

To further characterize the thermal degradation of the two disulfide-rich peptides, LC-MS analysis was performed to evaluate the degradation profiles of the heat-treated samples. As shown in Supplementary Figure S2, the LC-MS results were in good agreement with the HPLC data. Quantitative analysis indicated that ziconotide exhibited an apparent degradation of 11.8% ± 7.2% without centrifugation and 16.6% ± 0.01% following centrifugation. In contrast, linaclotide showed an apparent degradation of 15.5% ± 7.5% in the non-centrifuged samples and 6.7% ± 3.2% after centrifugation (Supplementary Figure S3).

While HPLC and LC-MS results provide a preliminary assessment of thermal degradation, it cannot resolve subtle conformational changes. Because separation relies primarily on hydrophobicity or polarity, conformational variants that do not alter these properties may coelute, yielding apparently unchanged peaks. Thus, HPLC and traditional LC-MS are intrinsically limited in detecting conformational heterogeneity, capturing only slight chemical degradation or pronounced structural alterations.

### 3.2 TOF-MS analysis of ziconotide and linaclotide

**FIGURE 3:**
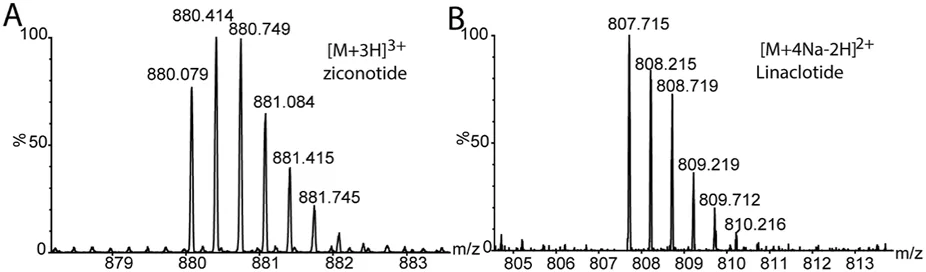
TOF mass spectrometric analysis of the molecular masses of ziconotide and linaclotide. **(A)** triply charged ions [M+3H]^3+^ (m/z 880.079) of ziconotide. **(B)** doubly charged sodium-adducted ions [M+4Na-2H]^2+^ (m/z 807.715) of linaclotide.

Direct infusion was employed to analyze ziconotide and linaclotide using time-of-flight mass spectrometry (TOF-MS), with the aim of identifying appropriate precursor ions for subsequent cyclic ion mobility mass spectrometry measurements. Untreated samples were subjected to full-scan analysis over the m/z range of 50–2000. As shown in Figure 3, under direct infusion conditions, ziconotide predominantly generated triply charged ions [M+3H]^3+^ (m/z 880.079) and quadruply charged ions [M+4H]^4+^ (m/z 660.308) (Figure 3A; Supplementary Figure S4), consistent with its theoretical molecular weight of 2639.13 Da.

In contrast, linaclotide was primarily observed as doubly charged sodium-adducted species under the same conditions (Figure 3), with [M+4Na-2H]^2+^ (m/z 807.715) being the dominant ion (Figure 3B; Supplementary Figure S4B). Interestingly, the singly charged linaclotide ion was barely detectable in the mass spectrum, showing only a trace signal, possibly because the compact conformation of linaclotide is unfavorable for ionization. When coupled with ion mobility separation, ziconotide remained predominantly protonated, whereas the pronounced sodium adduction observed for linaclotide is likely attributable to residual sodium ions introduced during the purification process. Notably, because these two peptides possess complex cyclic architectures stabilized by three pairs of disulfide bonds, further tandem mass spectrometric characterization using collision-induced dissociation (CID) provided only limited structural information (Supplementary Figure S5). Nevertheless, these results define the charge states and adduct distributions required for reliable cIM-MS measurements and further demonstrate that purification history and ionization conditions can significantly influence gas-phase ion chemistry and ion mobility behavior.

### 3.3 Instrument parameter tuning for cIM-MS analysis of ziconotide

We first optimized the cIM-MS parameters using ziconotide as a model analyte. In general, cIM-MS settings can be divided into two categories: global parameters and sequence-dependent parameters. Among the global parameters, the racetrack bias and the array offset during the separation phase exert the greatest influence on ion mobility separation. Racetrack bias defines the overall offset of the cIMS cell relative to the pre-transmission gradient potential and is the principal setting governing ion transmission. We therefore first optimized the racetrack bias. As shown in Supplementary Figure S6, ziconotide was separated under different racetrack bias values, and the best ion transmission was observed at a racetrack bias of 70 V.

During the cyclic ion mobility separation process, ions migrate from the array onto the racetrack. The array settings used in this phase have the strongest impact on ion transmission and are also most likely to generate artefactual peaks in cIMS experiments. We therefore further optimized the array offset using ziconotide. The results showed that when the array offset was set too low (50–60 V; Supplementary Figure S7), ziconotide ions could not be effectively separated, whereas values above 65 V enabled efficient separation of the generated ions. We speculate that an array offset set too low creates a potential gradient between the racetrack and the array, causing the array to act as an ion sink. Under these conditions, ions may be trapped by the array and then immediately expelled toward the detector upon transition from the separation phase to the eject-and-acquire phase. After optimization, an array offset of 70 V was also used for all subsequent experiments.

### 3.4 Optimization of instrument parameters for cIM-MS analysis of ziconotide and linaclotide

We further optimized the cIM-MS separation conditions for ziconotide to investigate the presence of previously overlooked topological isomers in the purchased batches of the active pharmaceutical ingredient. In cIM-MS, ions undergo multiple passes through a cyclic drift path, during which arrival time differences are cumulatively amplified with each cycle (Giles et al., 2019). Because subtly different conformers or isomers exhibit slight differences in mobility, increasing the number of passes (i.e., extending the effective drift time) progressively magnifies their arrival time separations, thereby enhancing resolving power. This unique feature enables cIM-MS to resolve minute conformational or topological differences in the gas phase with high stability (Giles et al., 2019). In principle, a compound existing as a single conformational or topological species would remain as a single sharp peak even after multiple passes through the cyclic drift region.

**FIGURE 4:**
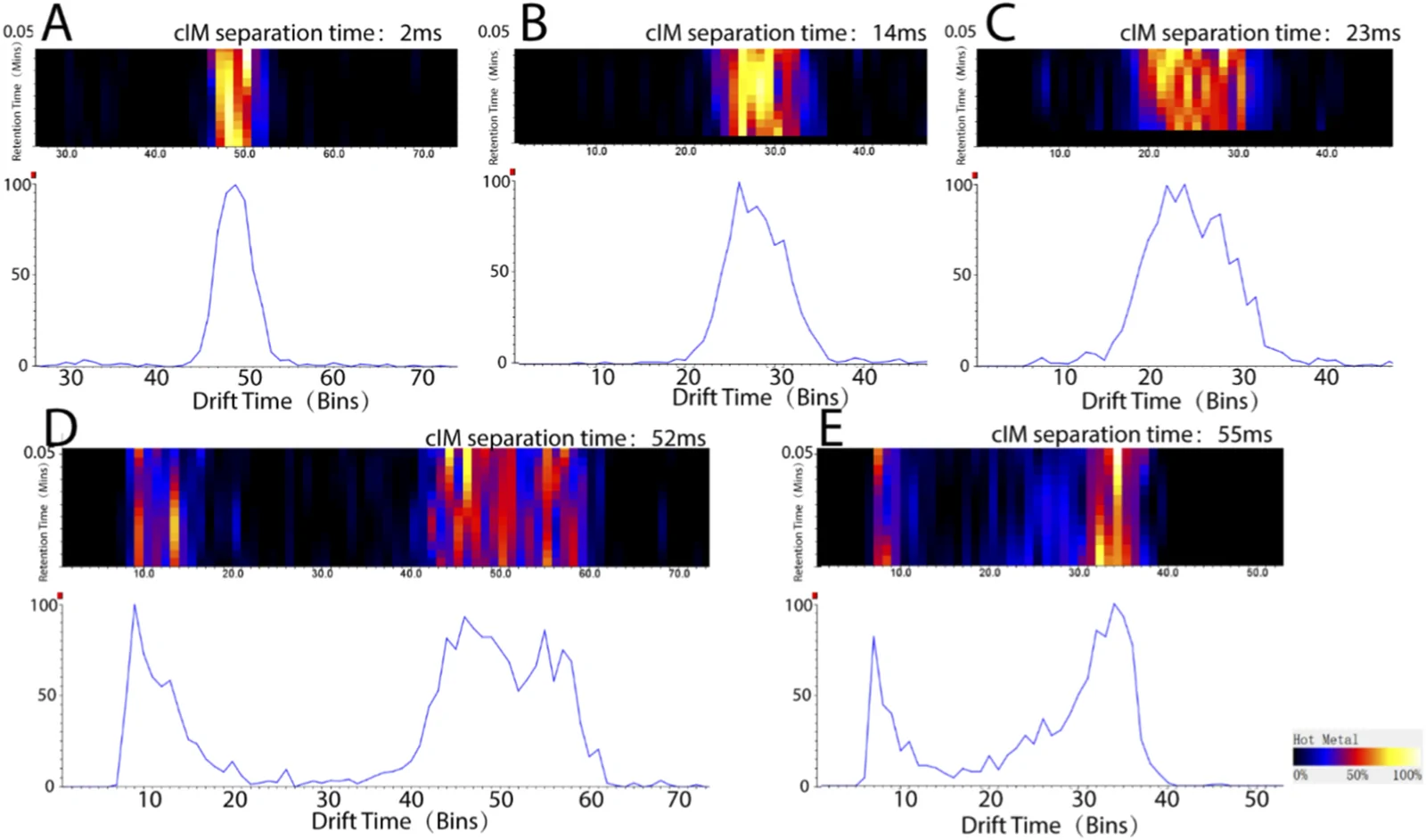
Effects of ion selection and drift time on the ion-mobility separation of ziconotide. Arrival time distributions of selected ions and their separation times: **(A)** [M+3H]^3+^, 2 ms; **(B)** [M+3H]^3+^, 14 ms; **(C)** [M+3H]^3+^, 23 ms; **(D)** [M+3H]^3+^, 52 ms; and **(E)** [M+4H]^4+^, 55 ms; The numbers ms denote the separation times used for data acquisition; Drift time refers to the time required for ions to traverse the mobility cell under the applied electric field. The heat maps are displayed using the Hot Metal color scale.

Guided by this principle, we performed mobility separation experiments on the triply protonated ziconotide ion ([M+3H]^3+^, m/z 880.079) at drift times of 2 ms, 14 ms, 23 ms, and 52 ms. As shown in Figure 4A, when ions completed only a single short pass (2 ms), a single, well-defined peak was observed. Upon extending the separation time to 14 ms (Figure 4B), multiple peaks emerged. Further prolongation to 23 ms (Figure 4C) resulted in clearer resolution of the topological isomers of ziconotide, revealing the structural complexity of the sample. These observations are consistent with our expectation that, owing to the intricate structure of ziconotide, multiple topological isomers coexist in the material. When the drift time was increased to 52 ms (Figure 4D), the majority of these topological isomers were effectively separated. To further validate these findings, we examined the quadruply protonated ion ([M+4H]^4+^, m/z 660.308). As shown in Figure 4E, although the arrival time distribution differed slightly from that of the triply charged species, the majority of topological isomers were again clearly resolved based on their distinct drift times.

In contrast to ziconotide, linaclotide is a smaller cyclic peptide consisting of only 14 amino acids, with a molecular weight of approximately 1,526 Da. Its cyclic structure is stabilized by three disulfide bonds, conferring relative stability in the gastrointestinal environment (Figure 1B). We hypothesized that its topological complexity would be lower than that of ziconotide.

**FIGURE 5:**
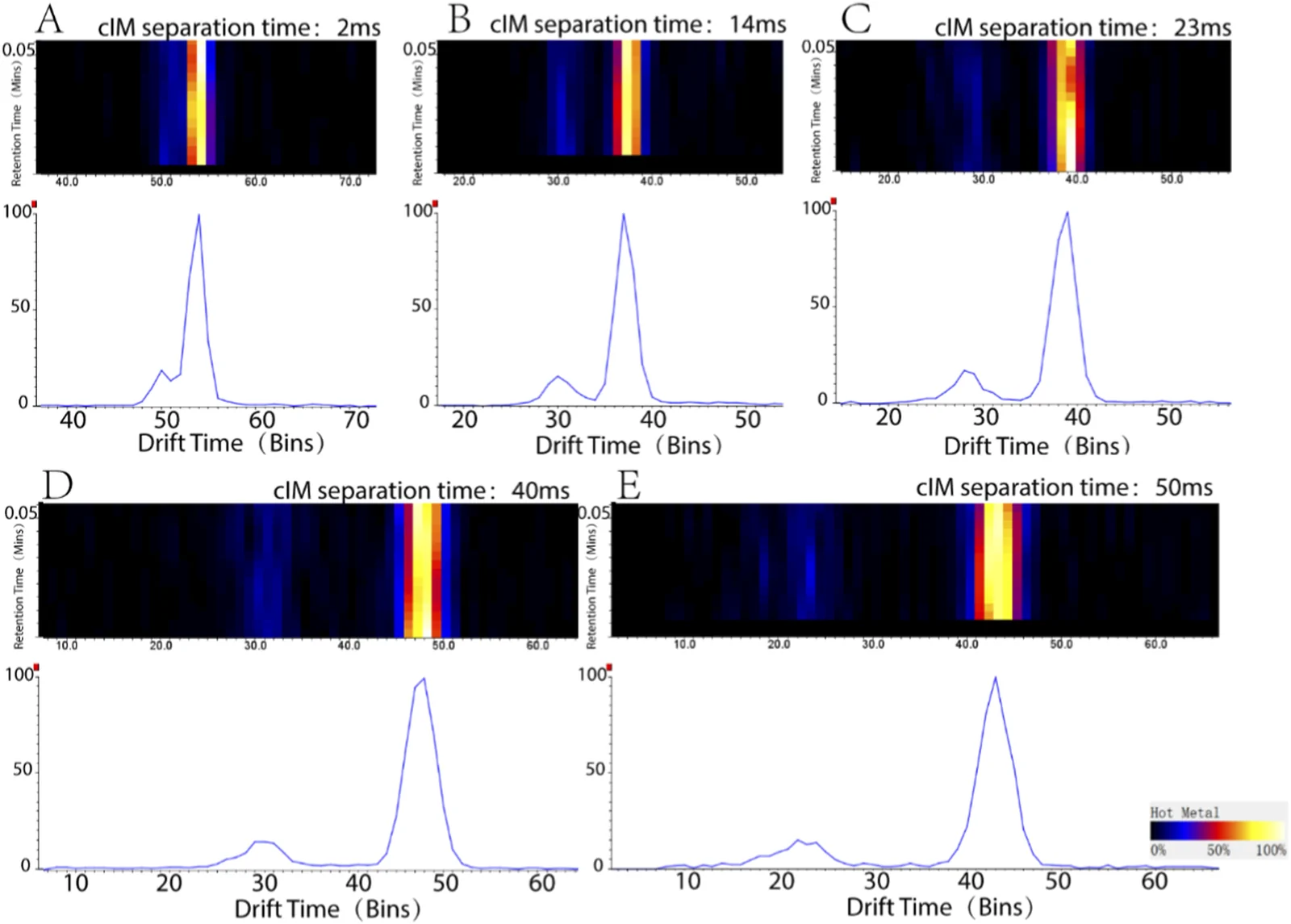
Effects of drift time on the ion-mobility separation of linaclotide. Arrival time distributions of selected ions and their separation times: **(A)** 2 ms; **(B)** 14 ms; **(C)** 23 ms; **(D) **40 ms; **(E)** 50 ms. The heat maps are displayed using the Hot Metal color scale.

We performed analogous mobility separation experiments on the [M+4Na-2H]^2+^ ion of linaclotide (m/z 807.715) at drift times of 2 ms, 14 ms, 23 ms, 40 ms, and 50 ms. As shown in Figure 5A, when ions underwent only a single short pass through the cyclic drift path (2 ms), a substantial population of unresolved topological isomers was observed. Upon extending the separation time to 14 ms, these topological isomers were clearly resolved. In marked contrast to ziconotide, linaclotide exhibited only a minor proportion of topological isomers in cIM-MS, with the dominant peak remaining highly homogeneous. Further increases in drift time to 23 ms, 40 ms, and 50 ms resulted in progressively improved resolution of the topological isomers. At a drift time of 40 ms used for ion-mobility separation, the calculated relative abundance of the isomer was approximately 18.55% ± 0.49% (Supplementary Figure S8). These results demonstrate that, by optimizing drift separation times, cIM-MS provides a powerful analytical tool for the precise characterization of topological isomers in cyclic peptides.

To further verify that the multiple mobility features observed in the ion mobility spectra originated from intrinsic structural variants of the peptide drugs rather than instrumental artifacts, we employed stlassin as a reference standard for cIM-MS validation. Stlassin is a ribosomally synthesized and post-translationally modified peptide (RiPP) produced by microorganisms that adopts a well-defined lasso topology (Liu et al., 2021). Its C-terminal tail is mechanically locked within the macrolactam ring by sterically bulky tryptophan residues, resulting in a unique and highly constrained three-dimensional structure. Consequently, stlassin is expected to exist predominantly as a single conformational species. The [M+2H+2Na]^4+^ ion of stlassin (m/z 870.975) was subjected to cyclic ion mobility analyses with mobility separation times of 5, 55, and 115 ms. In all cases, only a single mobility peak was observed, with no evidence of multiple structural variants (Supplementary Figure S9). These results provide supporting evidence that the multiple mobility features detected for ziconotide and linaclotide are attributable to intrinsic structural variants rather than artifacts arising from the cIM-MS measurement.

To further substantiate this hypothesis, ziconotide and linaclotide were treated with dithiothreitol (DTT) to reduce their disulfide bonds and disrupt their native tertiary structures prior to cyclic ion mobility analysis (Supplementary Figure S10). Following DTT treatment, the number of structural variants detected for both peptides decreased markedly (Supplementary Figure S11, Supplementary Figure S12). Most notably, linaclotide, which originally exhibited two distinct structural variants, displayed only a single mobility peak after reduction, indicating that the linearized peptide adopts a substantially more homogeneous gas-phase conformation. In contrast, although the number of structural variants observed for ziconotide was also reduced after DTT treatment, multiple mobility features were still detected. This behavior is likely attributable to its larger molecular size and longer amino acid sequence, which allow the linear peptide to adopt multiple gas-phase conformations despite the disruption of its native disulfide-bonded structure.

### 3.5 Enhanced characterization of peptide ion swertttubspecies via slice-based ion mobility selection

While conventional cyclic IM-MS provides an overall mobility-resolved distribution of ions after a single separation, the slice function allows ions within a selected arrival time window to be isolated and reinjected into the cyclic mobility device for further mobility separation. This tandem ion mobility (IM–IM) strategy enables peak-by-peak interrogation of individual conformers or topological isomers, facilitating the characterization of mobility-resolved ion subpopulations and revealing subtle conformational heterogeneity that may not be apparent from a single mobility separation.

**FIGURE 6:**
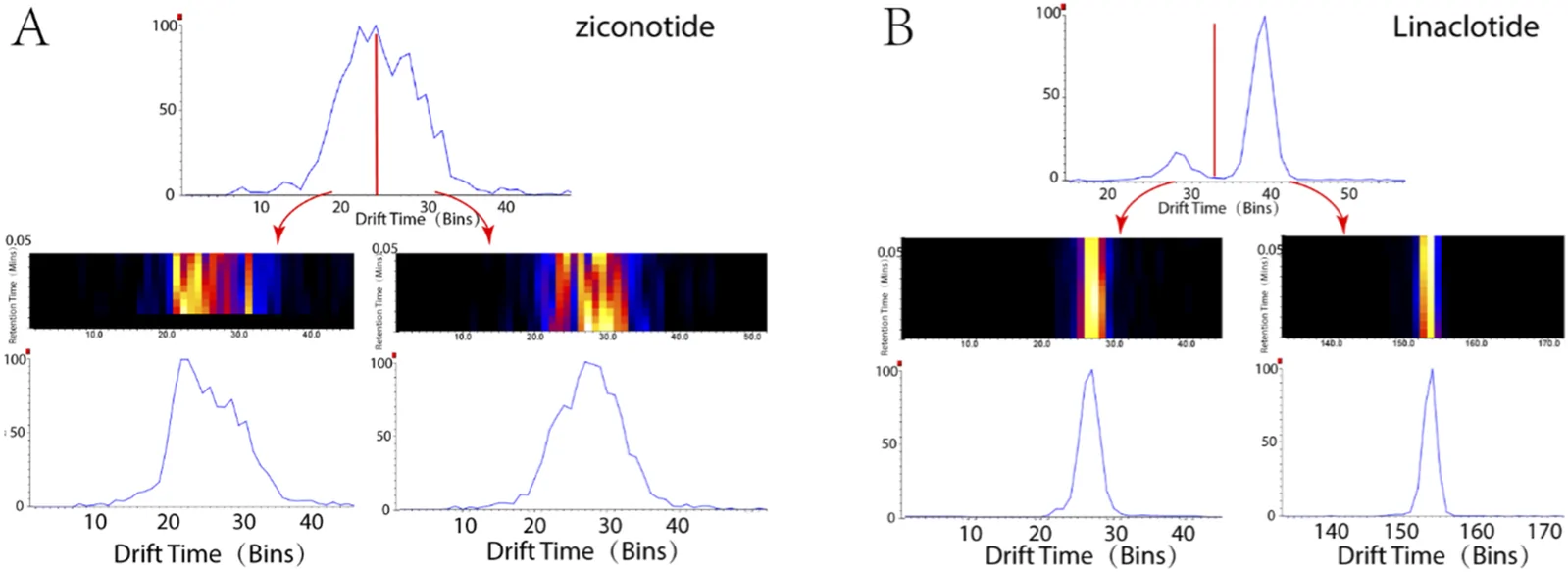
Analysis of peptide ion subspecies by slicing-based ion-mobility selection: **(A)** ziconotide and **(B)** linaclotide. The front and rear halves of the arrival time distributions of ziconotide and linaclotide after an initial 23 ms separation are shown.

To further interrogate the nature of the mobility-separated ions, we applied the slice function to the front and rear halves of the arrival time distributions of both ziconotide and linaclotide following an initial 23 ms separation. Each sliced subpopulation was then subjected to an additional 23 ms of mobility separation. As shown in Figure 6, both the leading and trailing halves of the ziconotide ion population exhibited pronounced conformational polymorphism. This may be due to the structural complexity of ziconotide; even after slicing by the slice technique, multiple structural variants still coexisted within each half. In striking contrast, the topological isomers of linaclotide, as well as its main peak, each resolved as a single sharp peak upon slicing, unambiguously confirming that they correspond to distinct topological isomers rather than rapidly interconverting conformers.

Collectively, these results demonstrate that the combination of cyclic ion mobility-mass spectrometry with the slice function not only achieves high-resolution separation of topological isomers in complex peptide molecules, but also enables fine dissection of conformational heterogeneity within individual ion populations. This approach provides a powerful analytical platform for detailed gas-phase structural characterization of cyclic peptides and other structurally intricate biomolecules.

### 3.6 Ion mobility fingerprint profiling reveals the thermal stability of the peptides

For decades, the quality assessment and control of biopharmaceuticals have lacked effective methods for the direct and intuitive evaluation of drug stability, quality changes, and degradation pathways. To address this gap, we explored the application of ion mobility fingerprint profiling based on cIM-MS to assess the degradation behavior of peptide drugs under accelerated thermal stress conditions.

**FIGURE 7:**
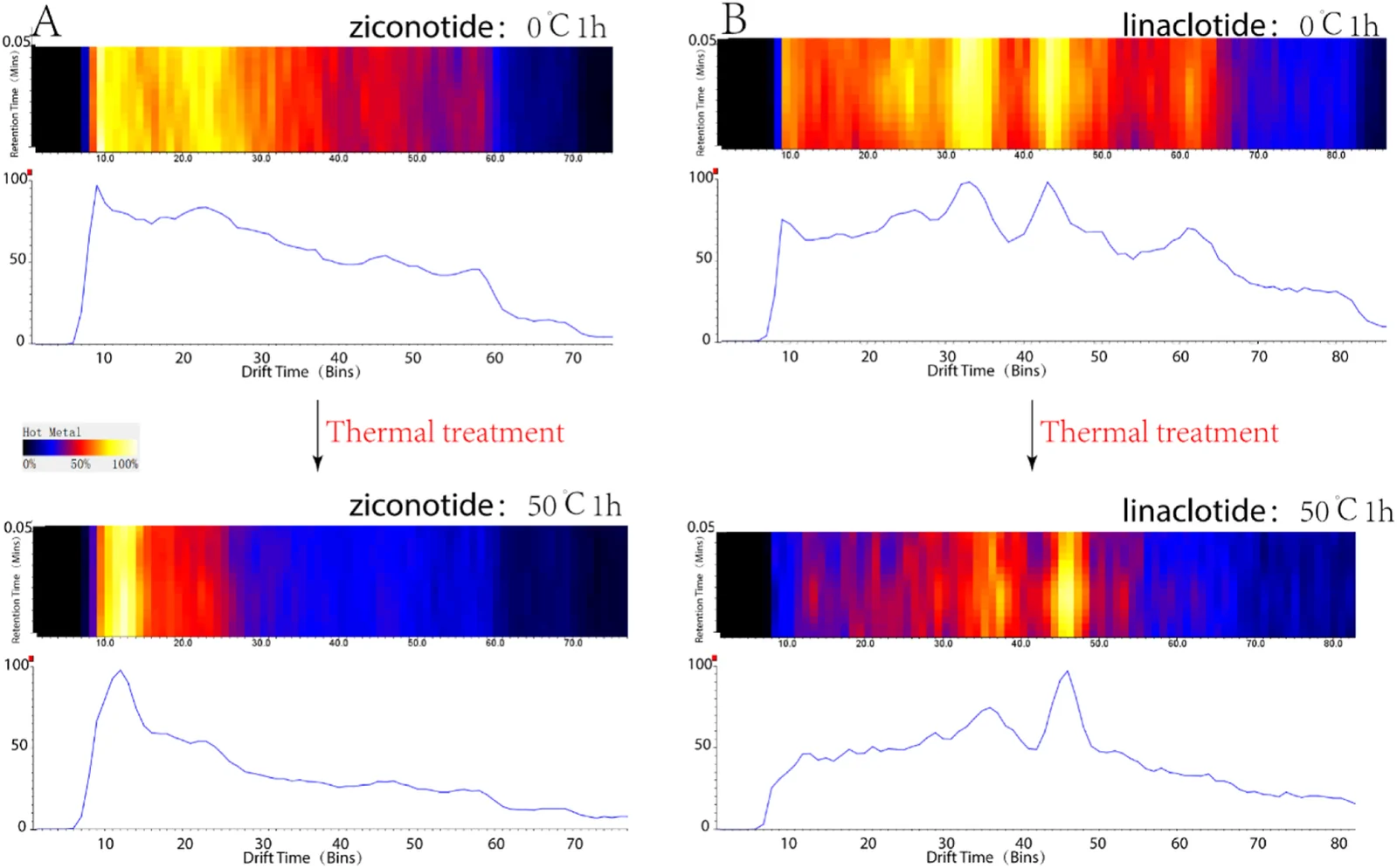
Ion-mobility fingerprint profiling of ziconotide **(A)** and linaclotide **(B)** following thermal treatment. The corresponding total ion chromatograms (TICs) are shown below the heatmap. The heat maps are displayed using the Hot Metal color scale.

We subjected the two model cyclic peptides, ziconotide and linaclotide, to heat treatment at 50 °C and 80 °C. Ion mobility fingerprint profiling was subsequently acquired for both compounds under a 50 ms mobility separation. As shown in Figure 7A, thermal treatment of ziconotide at 50 °C resulted in a substantial decrease in ion abundance, with the drift time versus retention time (dt/rt) heatmap revealing pronounced degradation. Similarly, the ion mobility fingerprint profiling of linaclotide indicated clear degradation under the same thermal stress conditions, albeit to a lesser extent compared with ziconotide. Upon treatment at 80 °C, both peptides underwent substantial degradation. The further 2D m/z–drift time (m/z-dt) mass spectra clearly revealed the degradation behavior of the two peptides as well as their conformational changes (Supplementary Figures S13-S14). This approach enables the intuitive and direct comparison of degradation profiles across samples, providing a powerful visual tool for the quality control and regulatory assessment of complex biopharmaceuticals.

**FIGURE 8:**
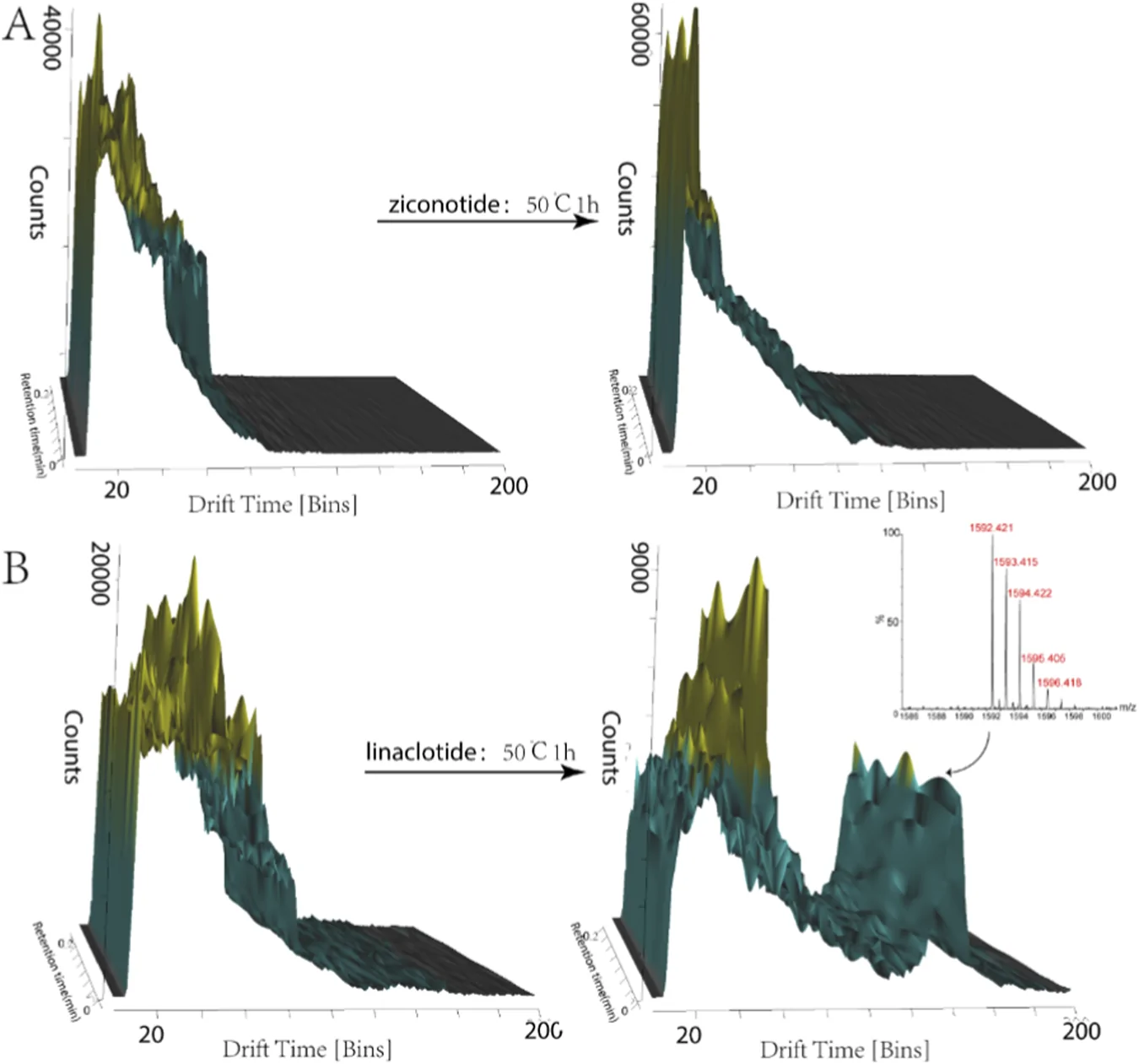
Three-dimensional mapping for ion mobility fingerprint profiling of ziconotide **(A)** and linaclotide **(B)** following thermal treatment (dt/rt).

Further three-dimensional imaging analysis of all ions in cIM-MS clearly visualizes the changes in major ion species within the samples. As shown in Figure 8A, ziconotide exhibited a marked reduction in ion abundance within the ∼40–60 bin region, accompanied by a substantial overall decrease in ion intensity, indicating pronounced degradation. Moreover, further insights confirmed that its conformation also underwent significant changes (Supplementary Figure S15, Supplementary Figure S16). In parallel, the ion distribution and intensity of linaclotide after 50 °C heat treatment also changed significantly, confirming a clear impact of thermal stress on sample integrity (Figure 8B). Notably, a distinct new ion peak emerged at approximately 140 bins in the heat-treated linaclotide sample. Subsequent mass spectrometric analysis revealed that these new peaks correspond primarily to singly charged ions at m/z 1548.418, 1570.438, 1592.421, and 1614.391, which form a series of multi-sodiated products derived from linaclotide (Supplementary Figure S17). Interestingly, only trace amounts of singly charged linaclotide ions were detected in the untreated sample. This suggests that linaclotide may undergo some conformational rearrangement. Upon unfolding, linaclotide may bind protons more readily, thereby facilitating the efficient formation of singly charged cations and leading to a marked enhancement of the signal. Notably, the mass spectrometric results differed markedly from those obtained by HPLC, highlighting the significant limitations of conventional HPLC in characterizing the quality of labile peptides.

**FIGURE 9:**
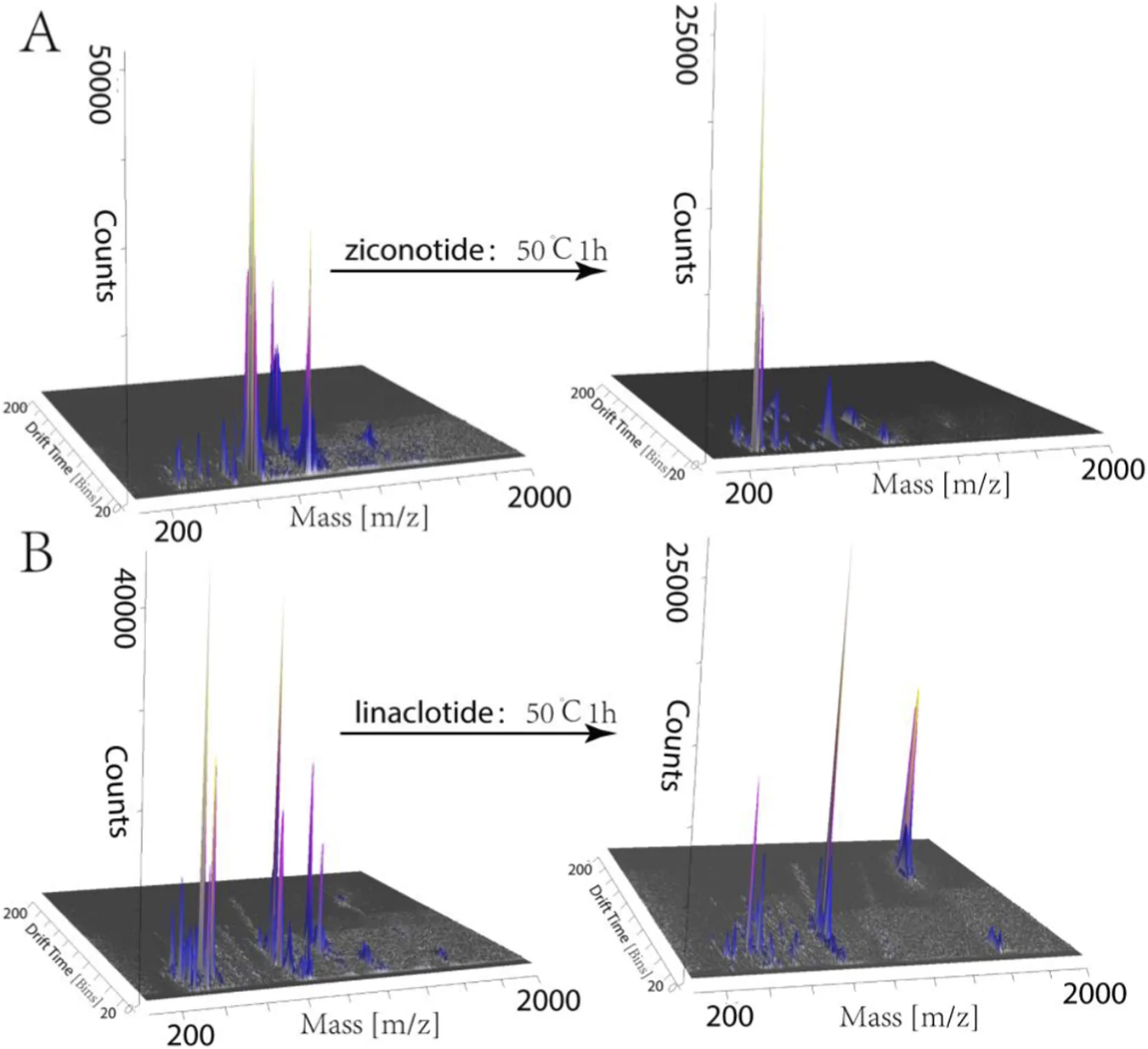
Three-dimensional mapping for ion mobility fingerprint profiling of ziconotide **(A)** and linaclotide **(B)** following thermal treatment (dt/ms).

Further three-dimensional m/z–drift time imaging analysis of all ions in cIM-MS clearly visualized ion degradation and conformational transitions (Figure 9). Ions with the same nominal mass underwent pronounced changes in both abundance and drift time, confirming that peptide drugs experience not only sequence degradation but also substantial conformational rearrangement during degradation. This approach thus precisely delineates the stress-induced fragmentation trajectories of the relevant peptide drugs.

**FIGURE 10:**
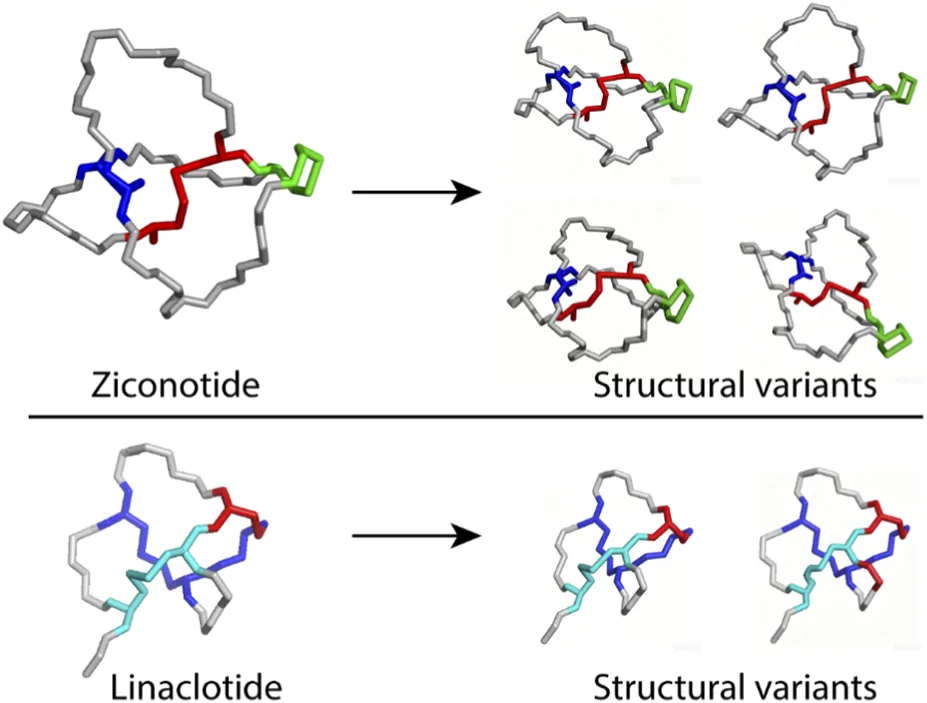
Hypothetical conformational variants of ziconotide and linaclotide. The illustrated structures represent possible three-dimensional conformations with identical primary sequences and disulfide-bond connectivity.

These observations suggest that multiple structural variants coexist in both native and thermally stressed samples of ziconotide and linaclotide. Although both peptides are constrained by three intramolecular disulfide bonds, their conformational ensembles may remain susceptible to subtle structural rearrangements, particularly during storage or under thermal stress. Such conformational distortions can give rise to multiple gas-phase structural variants while preserving the primary covalent structure (Figure 10). As peptide molecular size and structural complexity increase, this conformational heterogeneity is expected to become more pronounced, presenting significant analytical challenges for the quality assessment of disulfide-rich peptide therapeutics. These findings further highlight the need for conformation-sensitive analytical approaches capable of directly monitoring structural variants that cannot be resolved by conventional mass spectrometric methods alone.

Collectively, these results demonstrate that the three-dimensional imaging capability of cIM-MS, when combined with ion mobility fingerprint profiling, not only enables quantitative assessment of changes in ion abundance but also provides an intuitive visualization of degradation products. This integrated approach offers a powerful tool for the rapid and direct evaluation of stability and quality changes in biopharmaceuticals under accelerated stress conditions, thereby providing valuable methodological support for quality control and degradation mechanism studies of macromolecular drugs.

## 4 Discussion

Our results demonstrate that high-resolution cIM-MS can directly reveal the hidden topological heterogeneity of disulfide-rich peptide drugs. We found that ziconotide, a 25-residue cystine-knot peptide, exists in multiple stable conformations, whereas linaclotide (a 14-residue cyclic peptide) is largely homogeneous under native conditions. Such conformational polymorphism in a manufactured peptide drug has rarely been observed. The superior resolving power of cIM-MS was key to this discovery. By extending the effective drift time (number of passes) in the cyclic drift cell, we progressively amplified small mobility differences and separated coexisting conformers that would be indistinguishable by LC or conventional MS. This follows the general principle that subtle structural variants yield distinct IMS signals, and indeed we resolved multiple ziconotide peaks only when long drift times were employed. In contrast, linaclotide produced one dominant peak plus a minor isomeric peak, reflecting its simpler topology. These findings confirm our hypothesis that ziconotide’s dense cystine-knot network allows greater topological variety than linaclotide’s simpler fold.

The ion “slice” experiments provided further insight. Selecting early- and late-arriving ziconotide ions and re-separating them by mobility still yielded multiple peaks, indicating conformational heterogeneity within each sub-population. By contrast, sliced linaclotide sub-populations each yielded a single sharp peak, unambiguously showing that its two peaks represent discrete non-interconverting isomers, not rapid interconverting conformers. This clear partitioning validates that cIM-MS can isolate and probe single conformer ensembles from a mixture. In effect, the cIMS with slicing created a “conformational chromatography” in the gas phase, allowing us to dissect the structural complexity of each peptide peak.

The accelerated thermal-stress studies further illustrated the power of the cIMS fingerprint. Heating ziconotide led to a large drop in its overall ion signal, with the mobility heatmap (dt/rt plot) showing dramatic loss of the original peak region (∼40–60 bins). Linaclotide was more resilient: its main peak decreased less, but a new series of peaks appeared at longer drift times (∼140 bins), corresponding to singly charged fragments at m/z 1548–1614. These were identified as singly charged multi-sodiated products of linaclotide. The 3D IMS “fingerprints” clearly visualized these changes, effectively charting the emergence of specific degradation products and the loss of native structure. This capability has important practical implications. Conventional chromatographic or MS assays would detect peptide degradation only indirectly or after separation; in contrast, the cIMS fingerprint profiles directly map stability and impurity formation in a single ion mobility experiment. Such rapid visualization of sample degradation under stress provides a powerful tool for biopharmaceutical quality control.

Together, our findings establish cIM-MS as a spatially and conformationally resolved analytical platform for disulfide-rich peptide drugs. By adding an orthogonal drift-time dimension, it uncovers “hidden” related substances at the level of folding topology. For example, Cheng et al. showed that IMS could detect disulfide bond isomers of ziconotide; we now show that even without disulfide reshuffling, multiple native-like topologies can coexist. The ability to detect and monitor these species could greatly enhance impurity profiling and regulatory characterization of peptide therapeutics. In the broader context, this approach is readily generalizable to other complex peptides and protein drugs. Extending beyond conventional mass and sequence analysis, cIM-MS enables detailed mapping of conformational states and degradation pathways, thereby advancing our understanding of how peptide topology influences function and stability.

In summary, cIM-MS provides an unprecedented “conformational fingerprint” for complex peptide molecules. By revealing structural heterogeneity that would otherwise go unnoticed, this method adds a new dimension to analytical quality assessment. We anticipate that incorporating cIM-MS into peptide drug development and manufacturing will improve detection of topological impurities, guide formulation of stable products, and ultimately contribute to safer and more effective biopharmaceuticals.

## Data Availability

The raw data supporting the conclusions of this article will be made available by the authors, without undue reservation.
