# Supplementary material for: Resolving conformational polymorphism in disulfide-rich peptide drugs
Source: Front Chem. 2026 Aug 17;14:1913741. doi: 10.3389/fchem.2026.1913741 (PMC13526816; doi:10.3389/fchem.2026.1913741)
Supplement: Supplementary file 1 [file DataSheet1.pdf]

Supplementary Material

Resolving Conformational Polymorphism in Disulfide-Rich Peptide Drugs

Shaozhou Zhu[a], Yifeng Ge[b], Haiwei Huang[a,*] and Mingzhe Xu[a,*]

[a]National Institutes for Food and Drug Control, Beijing 102629, China

[b]Beijing Institute of Petrochemical Technology, Beijing 102617, China

*Correspondence

Haiwei Huang, E-mail: huanghw@nifdc.org.cn

Mingzhe Xu, E-mail: xumzhe@nifdc.org.cn

1 Supplementary Tables

Table S1. Instrument parameters used for cyclic IM-MS analysis.

Module	Parameter	Value
TOF-MS	Ionization mode	Positive ESI
	Scan range	*m/z* 50–2000
	Capillary voltage	3.0 kV
	Cone voltage	40 V
	Source temperature	150 °C
	Desolvation temperature	280 °C
	Desolvation gas flow	800 L h^{-1}

Module	Parameter	Value
Sample introduction	Flow rate	5 µL min^{-1}
Cyclic IMS	Racetrack bias	70 V
	Array offset	2 V
	Traveling wave velocity	375 m s^{-1}
	Traveling wave height	4 V
	Helium cell bias	20 V
Collision cell	Trap CE	6 V
	Transfer CE	4 V

2 Supplementary Figures

2.1 Supplementary Figures

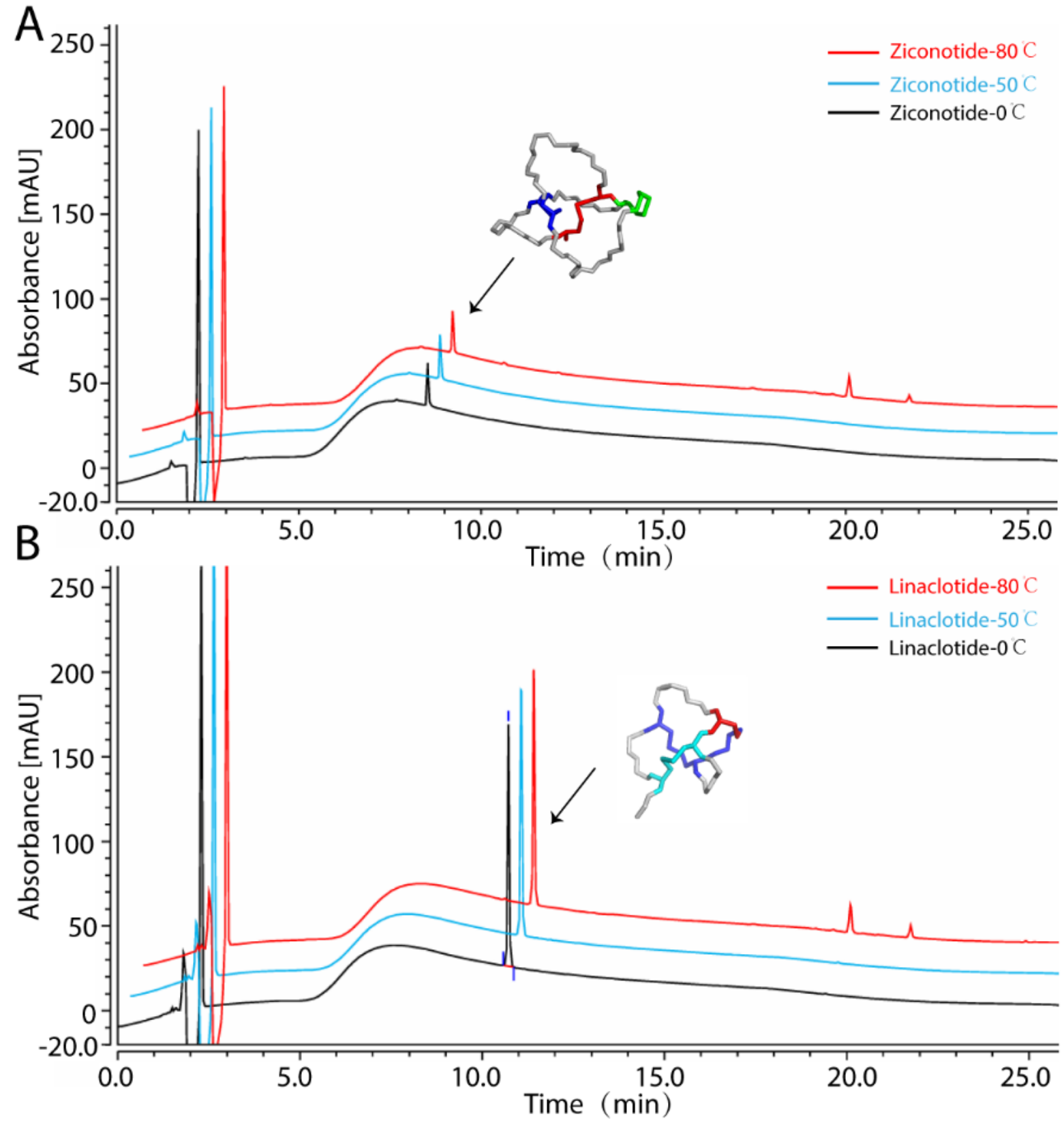

Figure S1. Temperature-dependent C18 HPLC profiles of ziconotide and linaclotide after heat treatment without centrifugation. (A) ziconotide; (B) linaclotide.

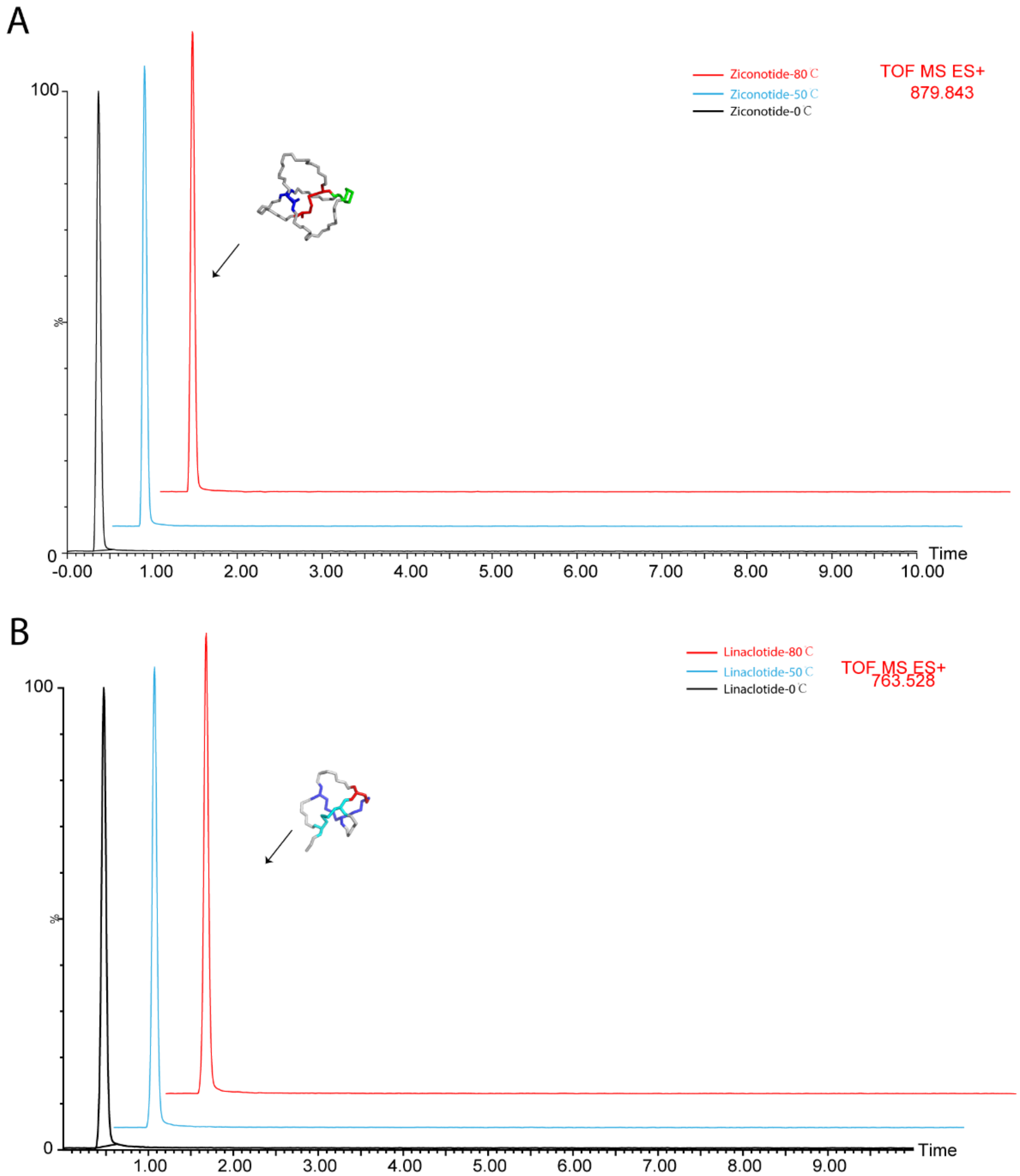

Figure S2. Temperature-dependent LC-MS profiles of ziconotide and linaclotide after heat treatment without centrifugation. (A) ziconotide; (B) linaclotide.

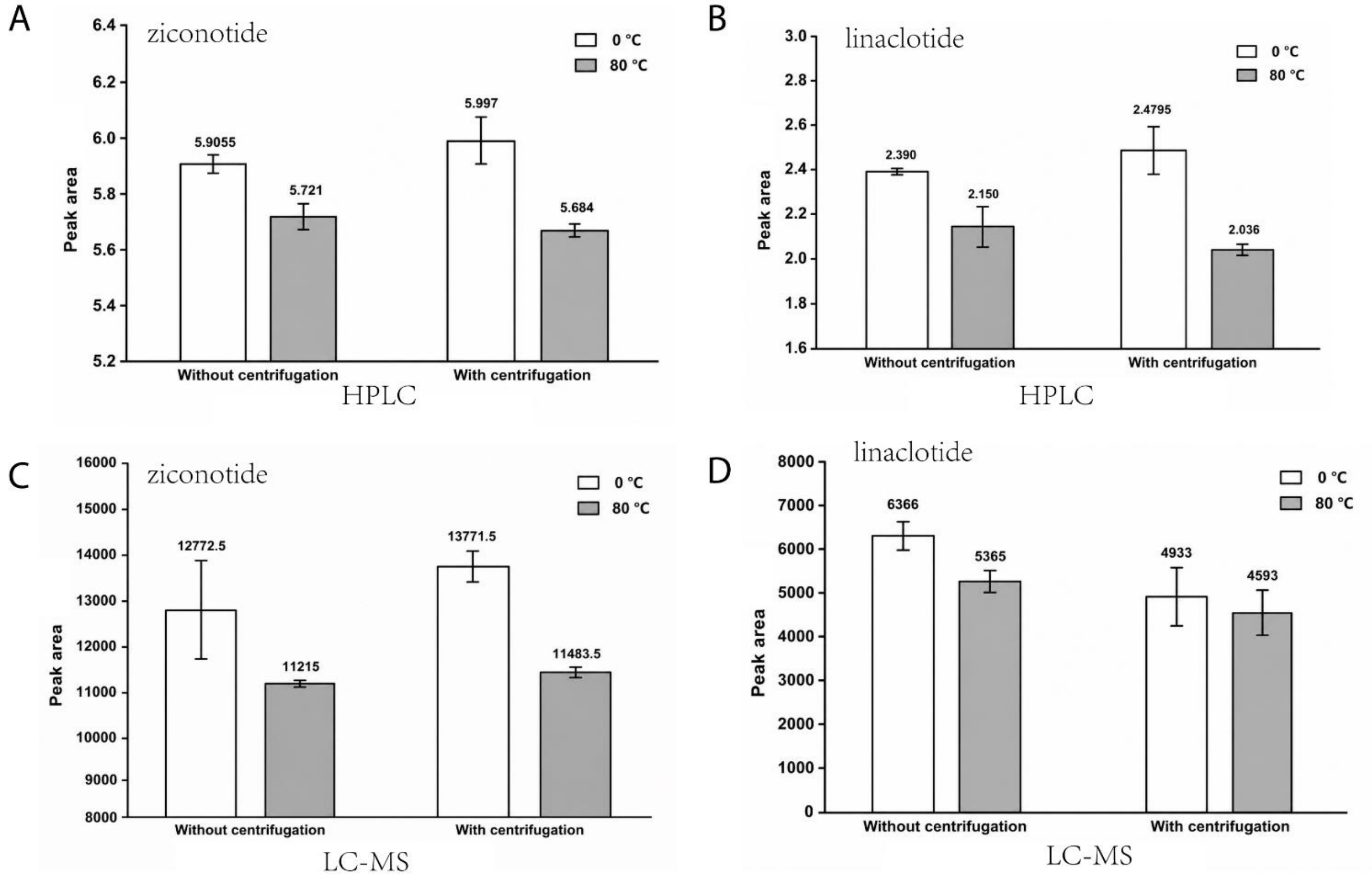

Figure S3. Comparison of peak areas of ziconotide and linaclotide before and after heat treatment at 80 °C. (A) and (B) HPLC peak areas. (C) and (D) LC–MS peak areas. Data are presented as mean ± SD (n = 2).

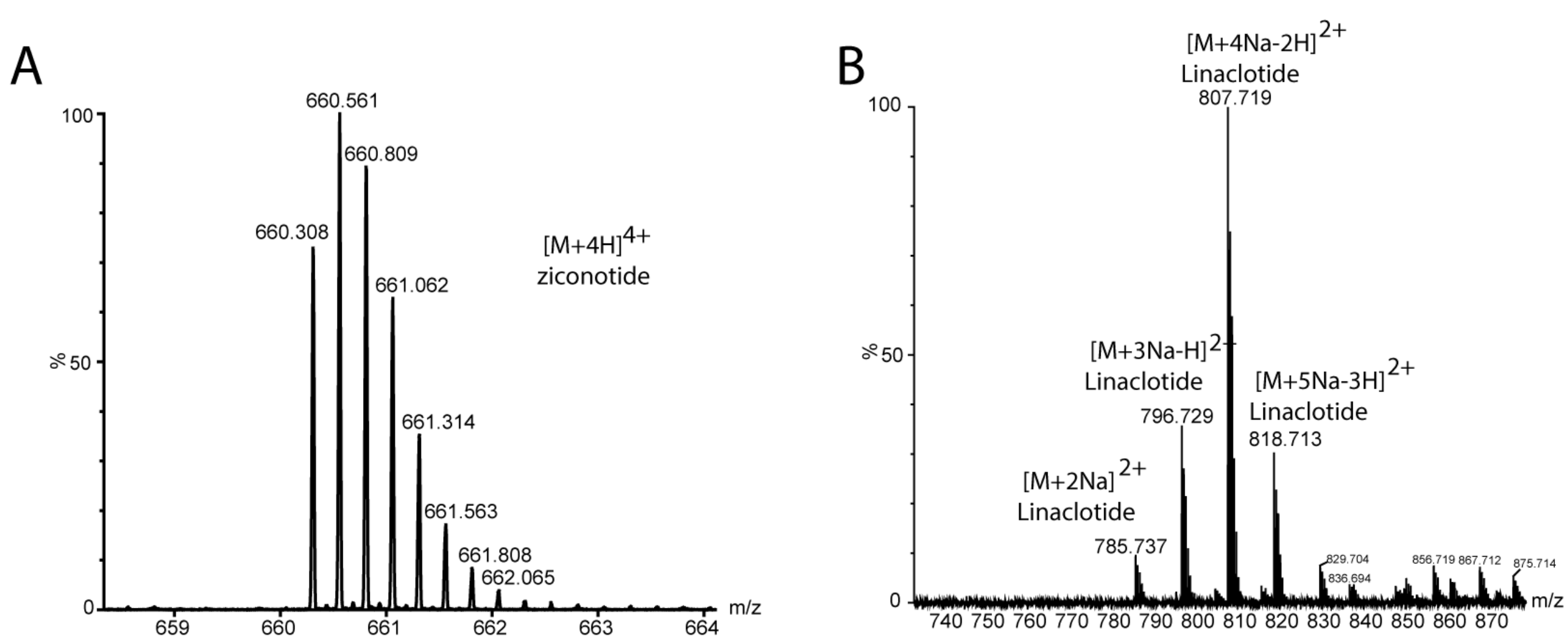

Figure S4. TOF mass spectrometric analysis of the molecular masses of ziconotide and linaclotide. (A) Ziconotide $[M+4H]^{4+}$ ion (m/z 660.308, theoretical m/z 660.282; mass deviation: 39.4 ppm). (B)

Doubly charged sodium-adducted ions of linaclotide, [M+4Na-2H]$^{2+}$ (m/z 807.715, theoretical m/z 807.666; mass deviation: 60.5 ppm).

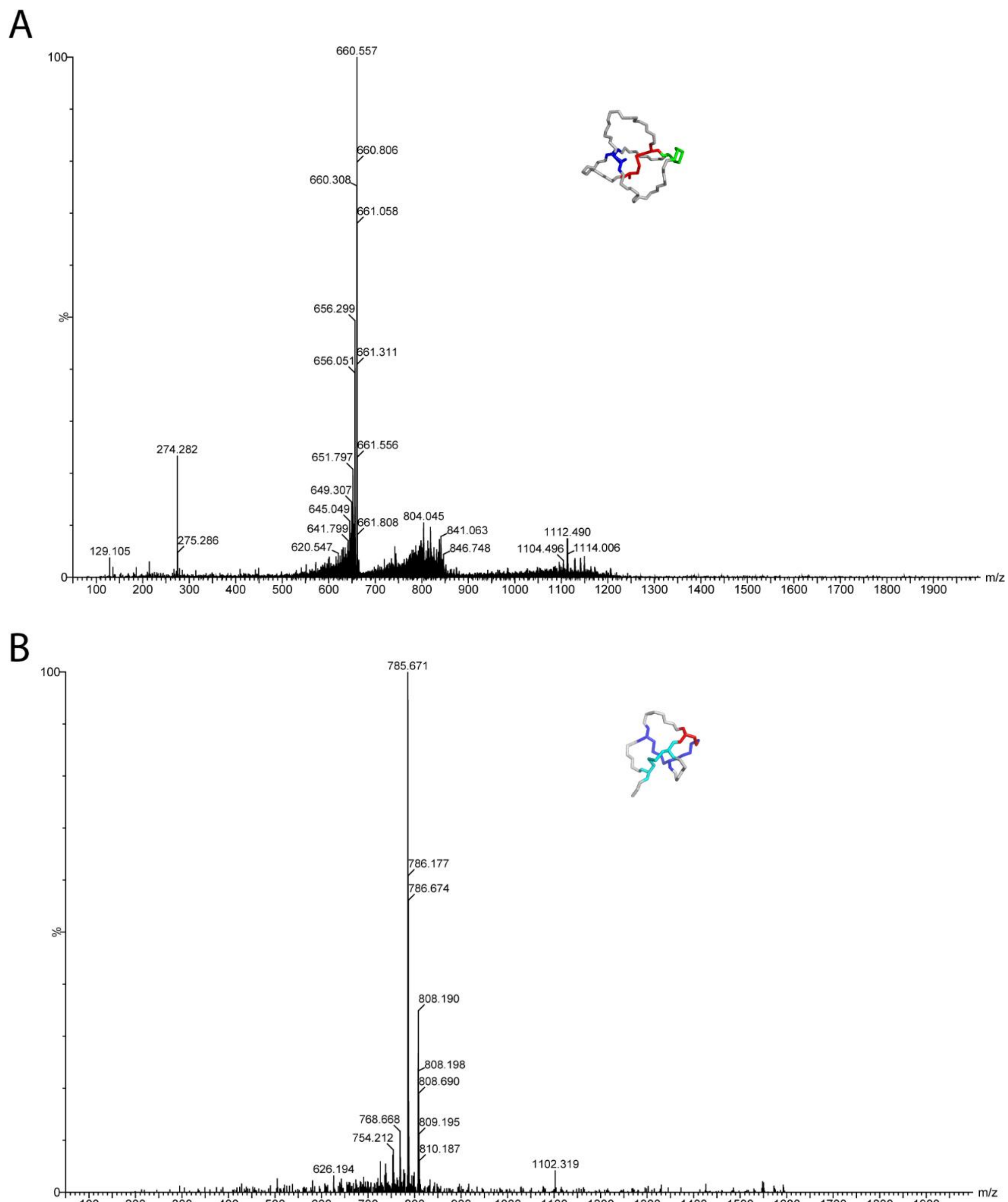

Figure S5. MS^2 spectra of ziconotide and linaclotide.

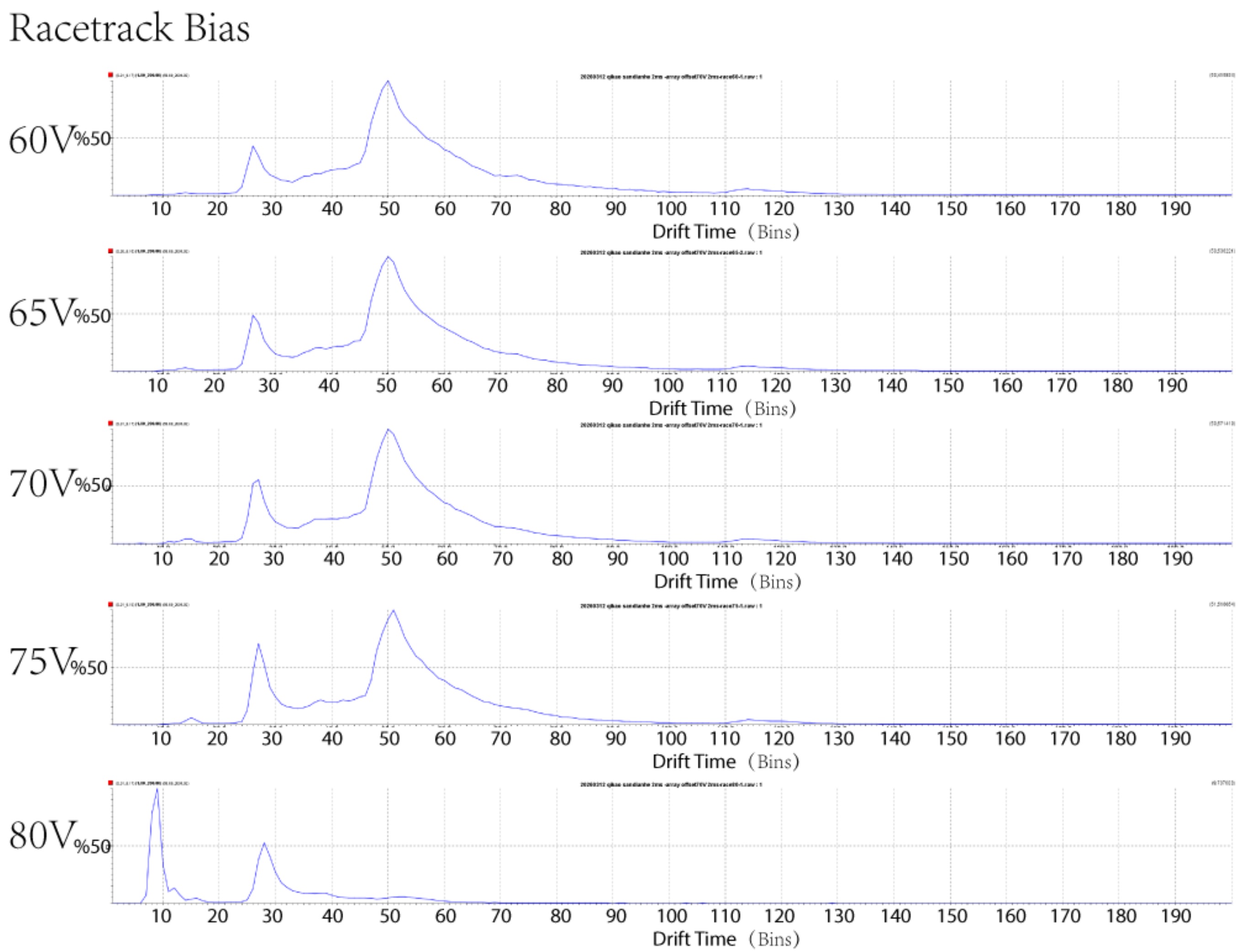

Figure S6. Effects of racetrack bias on the transmission of ziconotide. All other settings not being tested were kept at the values stated in the method section.

Array Offset

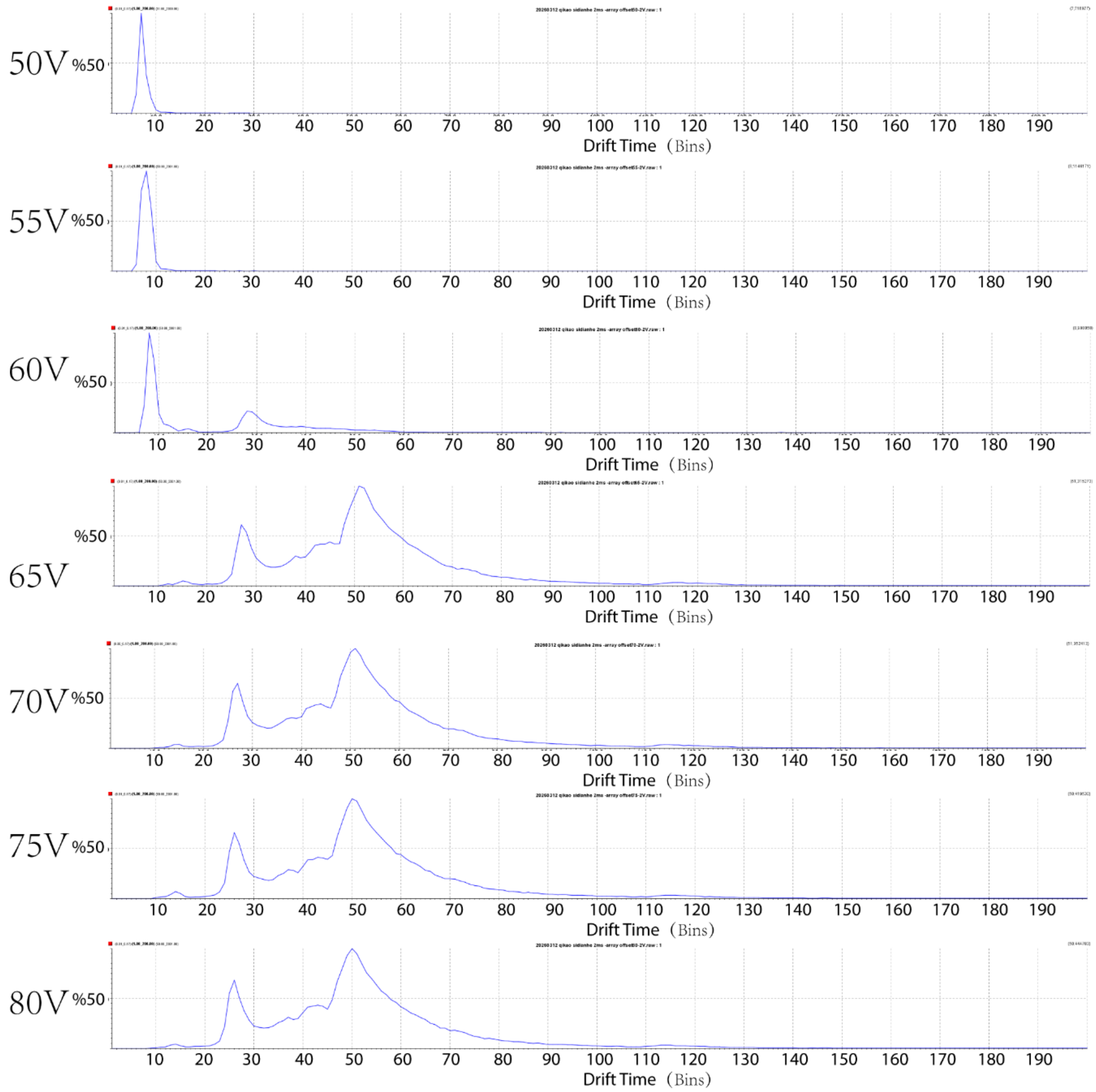

Figure S7. Effects of array offset voltage on the transmission of ziconotide. All other settings not being tested were kept at the values stated in the method section.

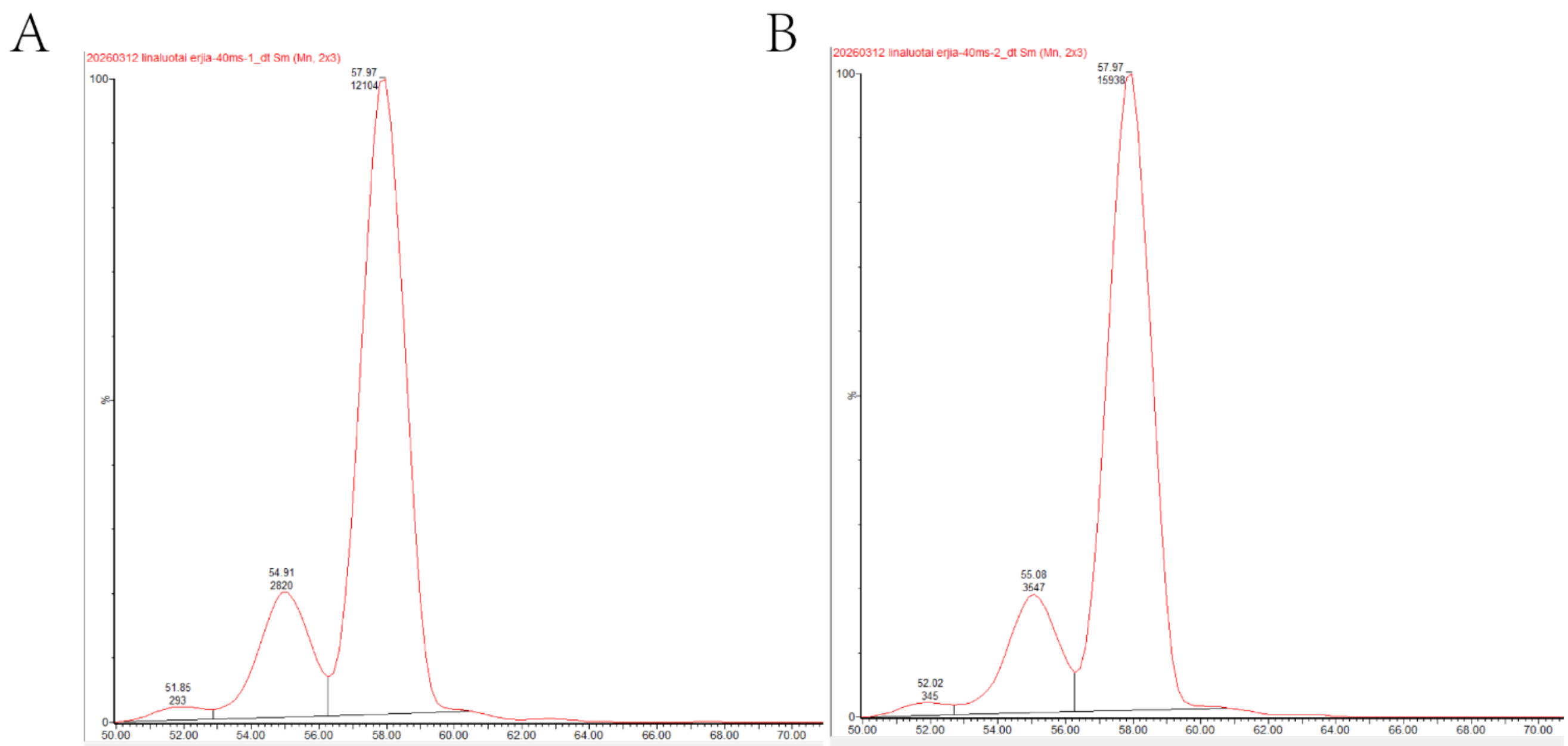

Figure S8 Quantitative analysis of the proportion of linaclotide topoisomers; experiments were performed in duplicate.

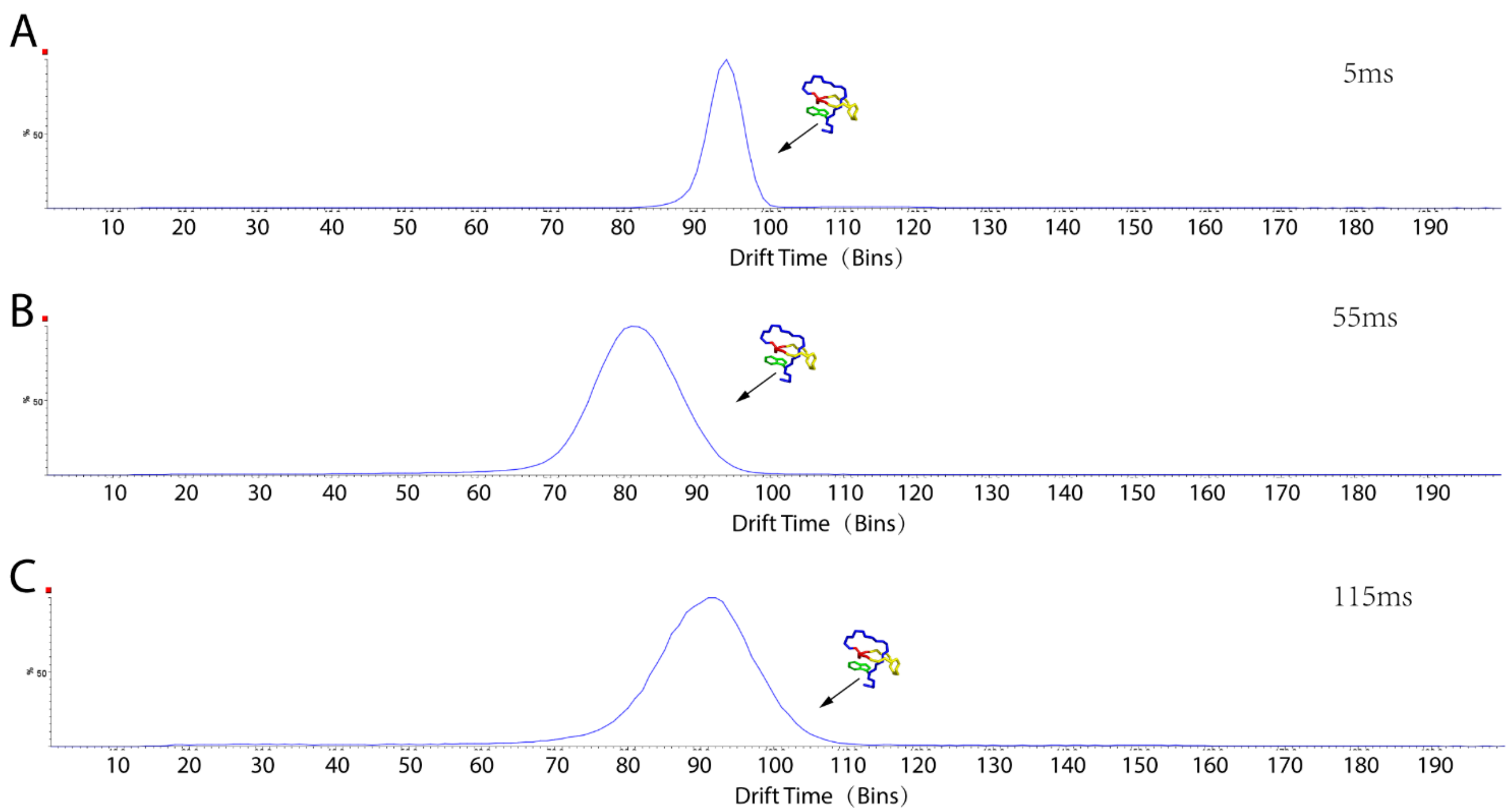

Figure S9 Effects of drift time on the ion-mobility separation of Stlassin. Arrival time distributions of selected ions and their separation times: (A) $[M+2H+2Na]^{4+}$, 5 ms; (B) $[M+2H+2Na]^{4+}$, 55 ms; (C) $[M+2H+2Na]^{4+}$, 115 ms

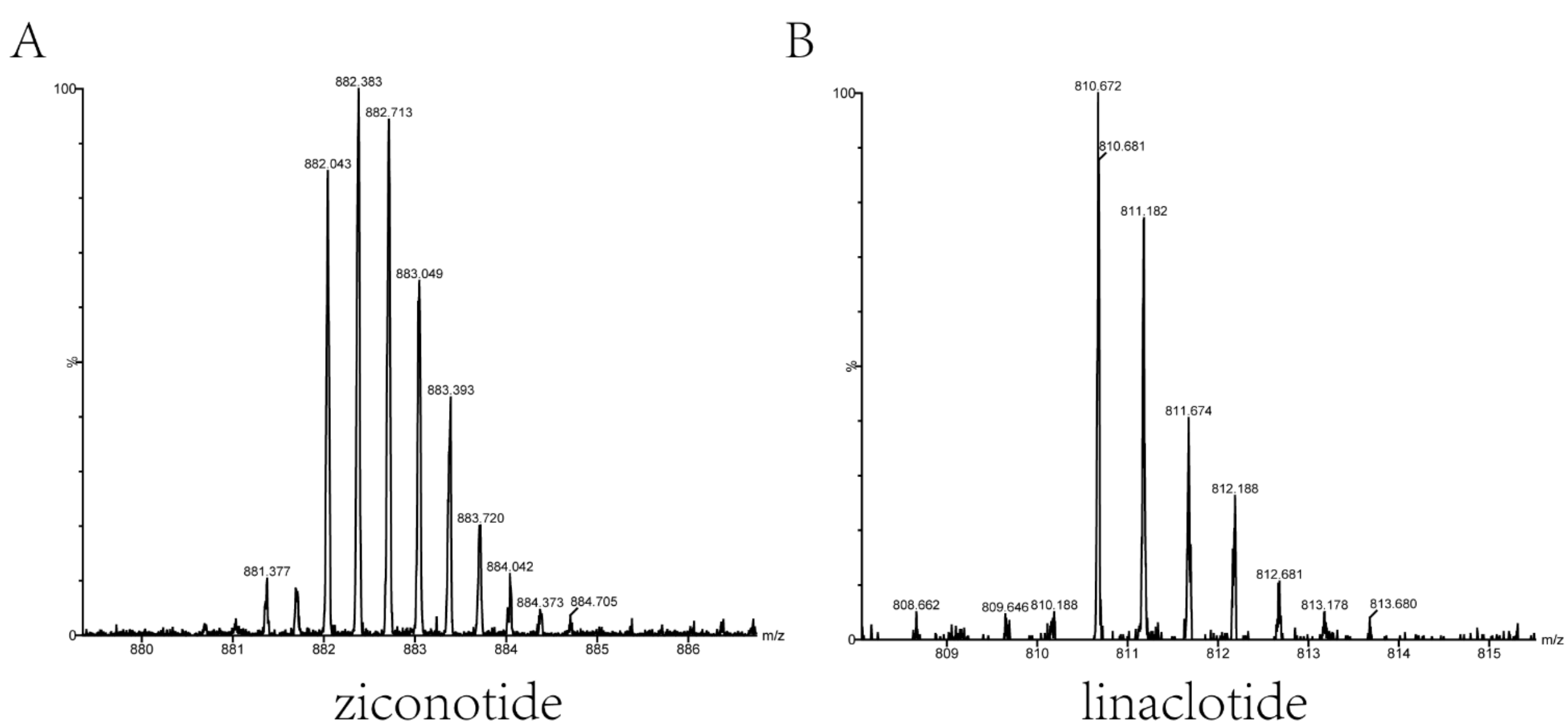

Figure S10 TOF mass spectrometric analysis of the molecular masses of ziconotide and linaclotide after treated with DTT. (A) Ziconotide [M+3H]$^{4+}$ ion (m/z 882.043). (B) doubly charged sodium-adducted ions[M+4Na-2H]$^{2+}$ (m/z 810.672) of linaclotide.

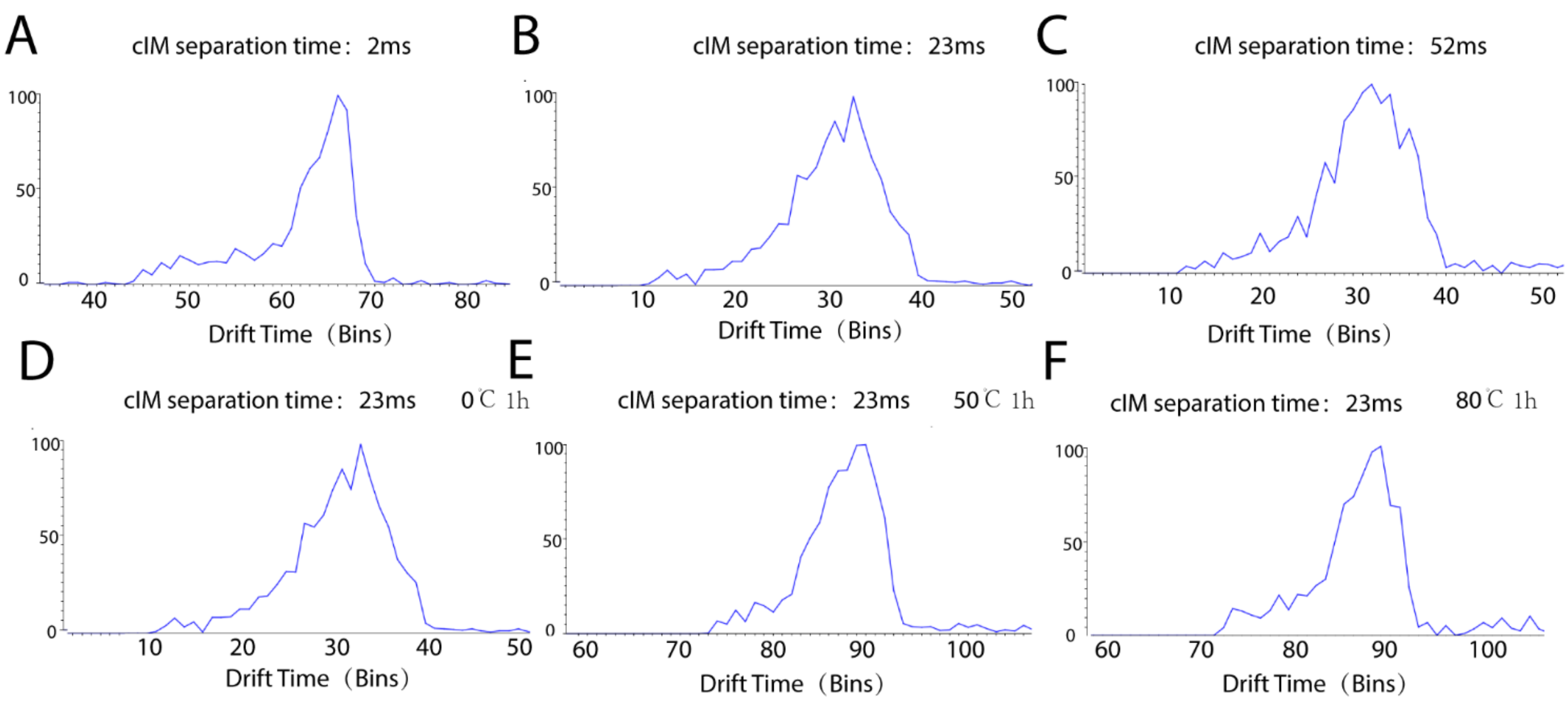

Figure S11 Effects of drift time and heat treatment on the ion-mobility separation of ziconotide. Arrival time distributions of selected ions and their separation times: (A) [M+3H]$^{4+}$, 2 ms; (B) [M+3H]$^{4+}$, 23 ms; (C) [M+3H]$^{4+}$, 52 ms; (D) [M+3H]$^{4+}$, 23 ms，0℃1h; (E) [M+3H]$^{4+}$, 23 ms, 50℃1h; (F) [M+3H]$^{4+}$, 23 ms, 80℃1h.

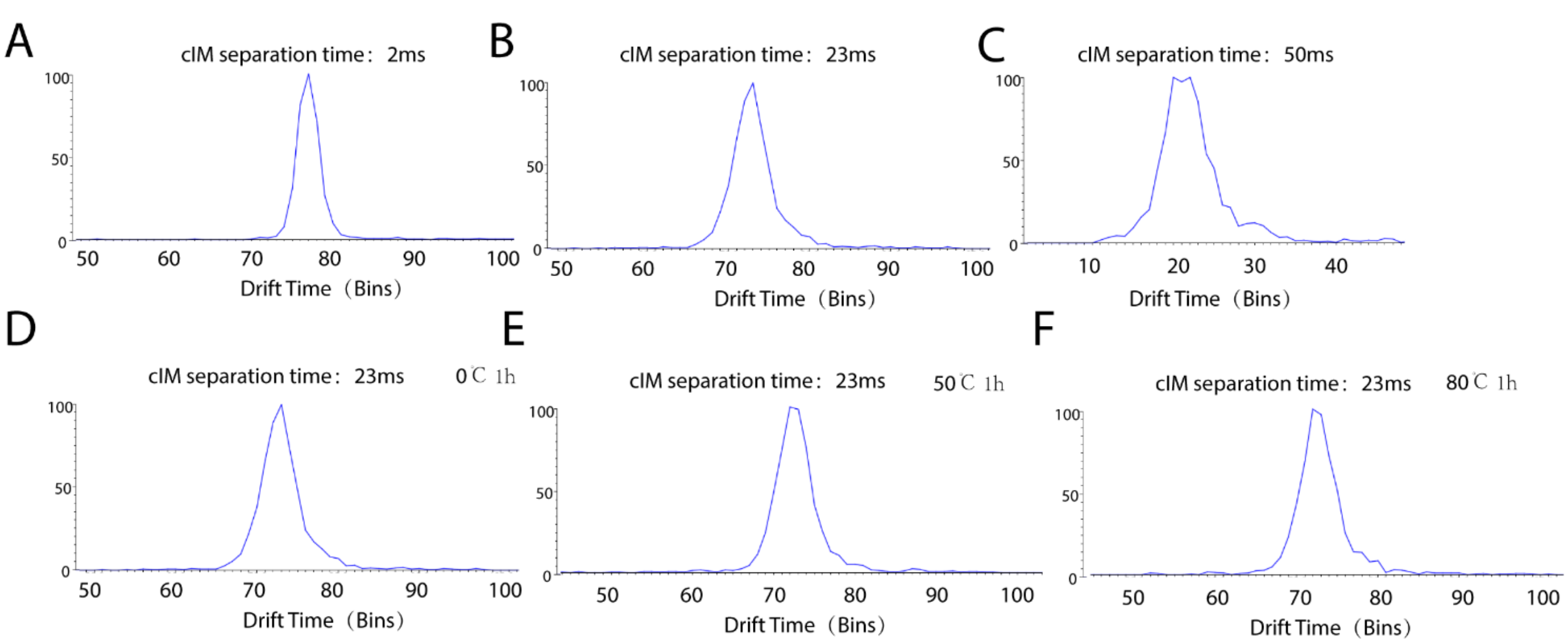

Figure S12 Effects of drift time and heat treatment on the ion-mobility separation of linaclotide. Arrival time distributions of selected ions and their separation times: (A) [M+4Na-

2H]$^{2+}$, 2 ms; (B) [M+4Na-2H]$^{2+}$, 23 ms; (C) [M+4Na-2H]$^{2+}$, 50 ms; (D) [M+4Na-2H]$^{2+}$, 23 ms, 0℃1h; (E) [M+4Na-2H]$^{2+}$, 23 ms, 50℃1h; (F) [M+4Na-2H]$^{2+}$, 23 ms, 80℃1h.

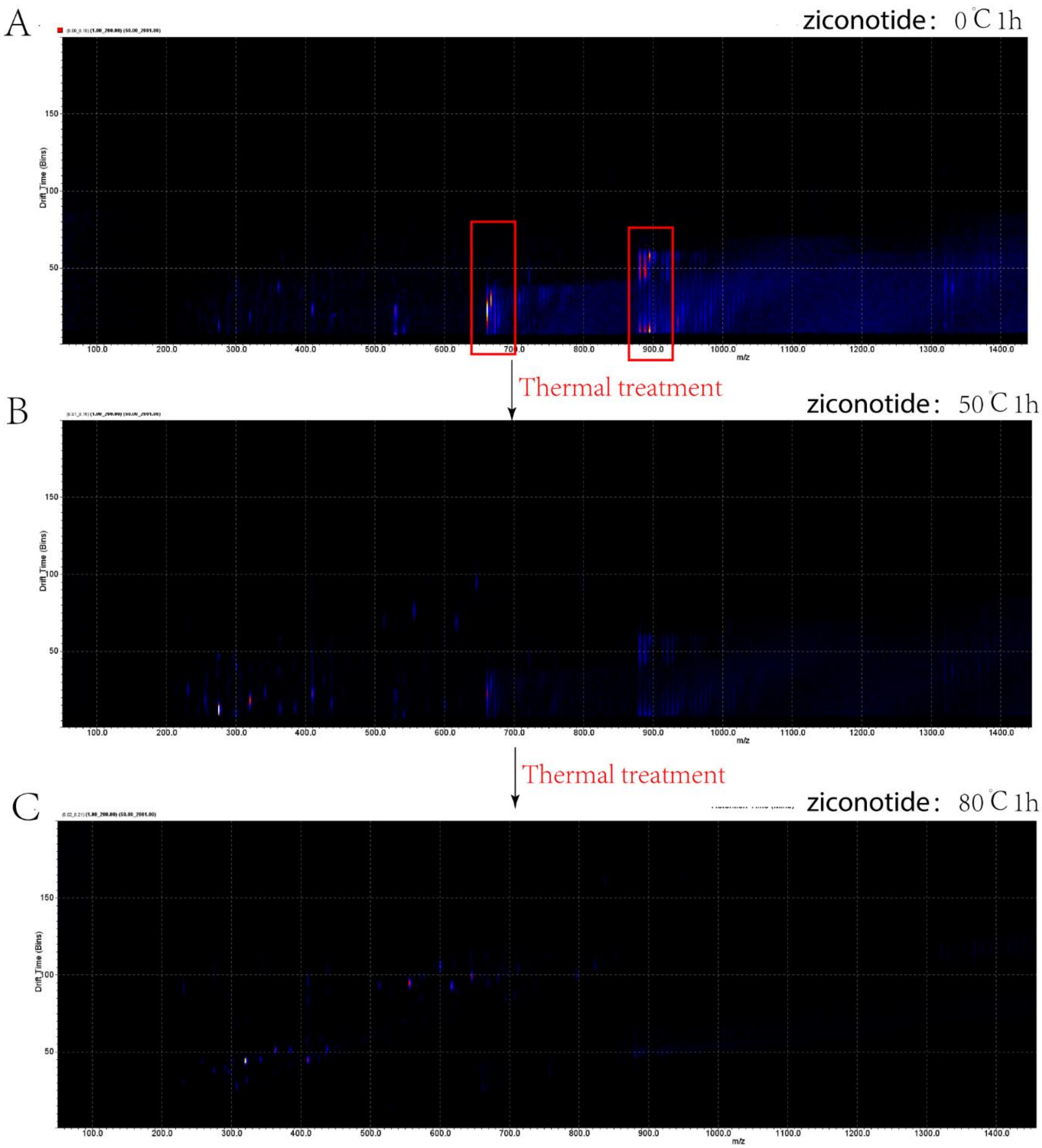

Figure S13 Ion-mobility fingerprint profiling of ziconotide following thermal treatment (ms/dt). (A) ziconotide:0℃1h；(B) ziconotide:50℃1h；(C) ziconotide:80℃1h

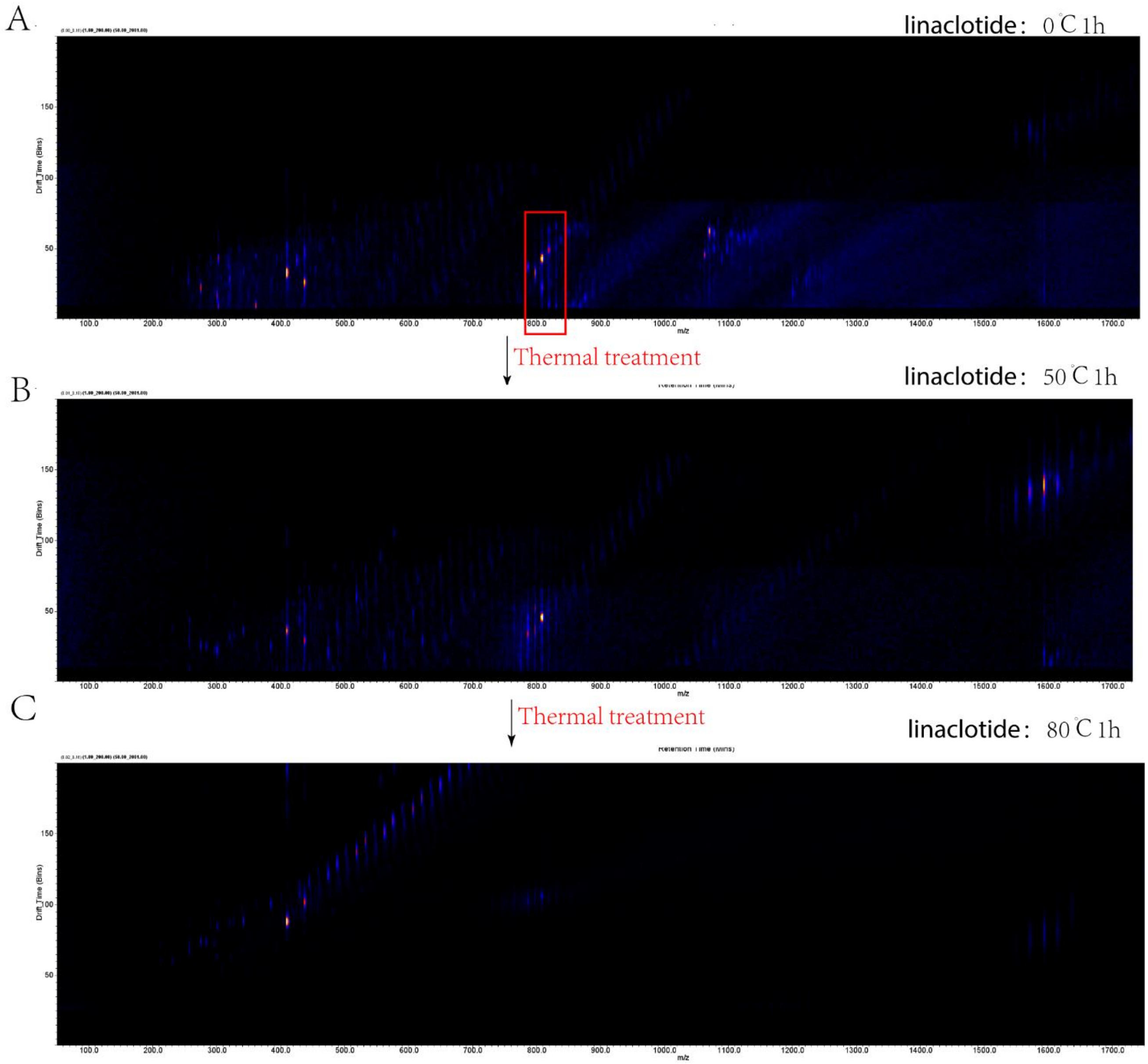

Figure S14 Ion-mobility fingerprint profiling of Linaclotide following thermal treatment (ms/dt). (A) Linaclotide:0℃1h；(B) Linaclotide:50℃1h；(C) Linaclotide:80℃1h

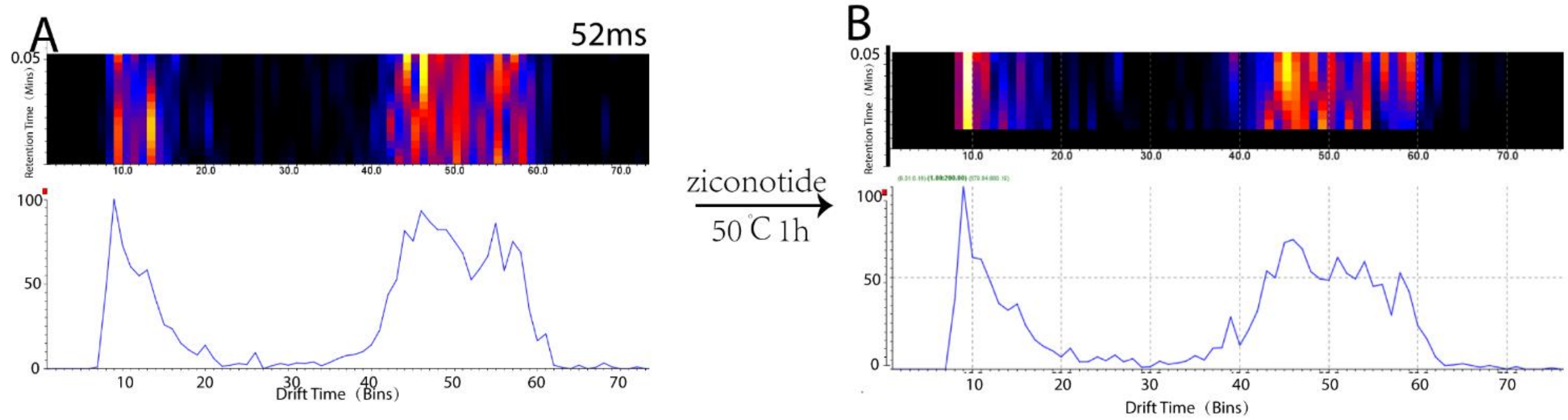

Figure S15 Effects of heat treatment on ziconotide topology monitored via $[M+3H]^{3+}$

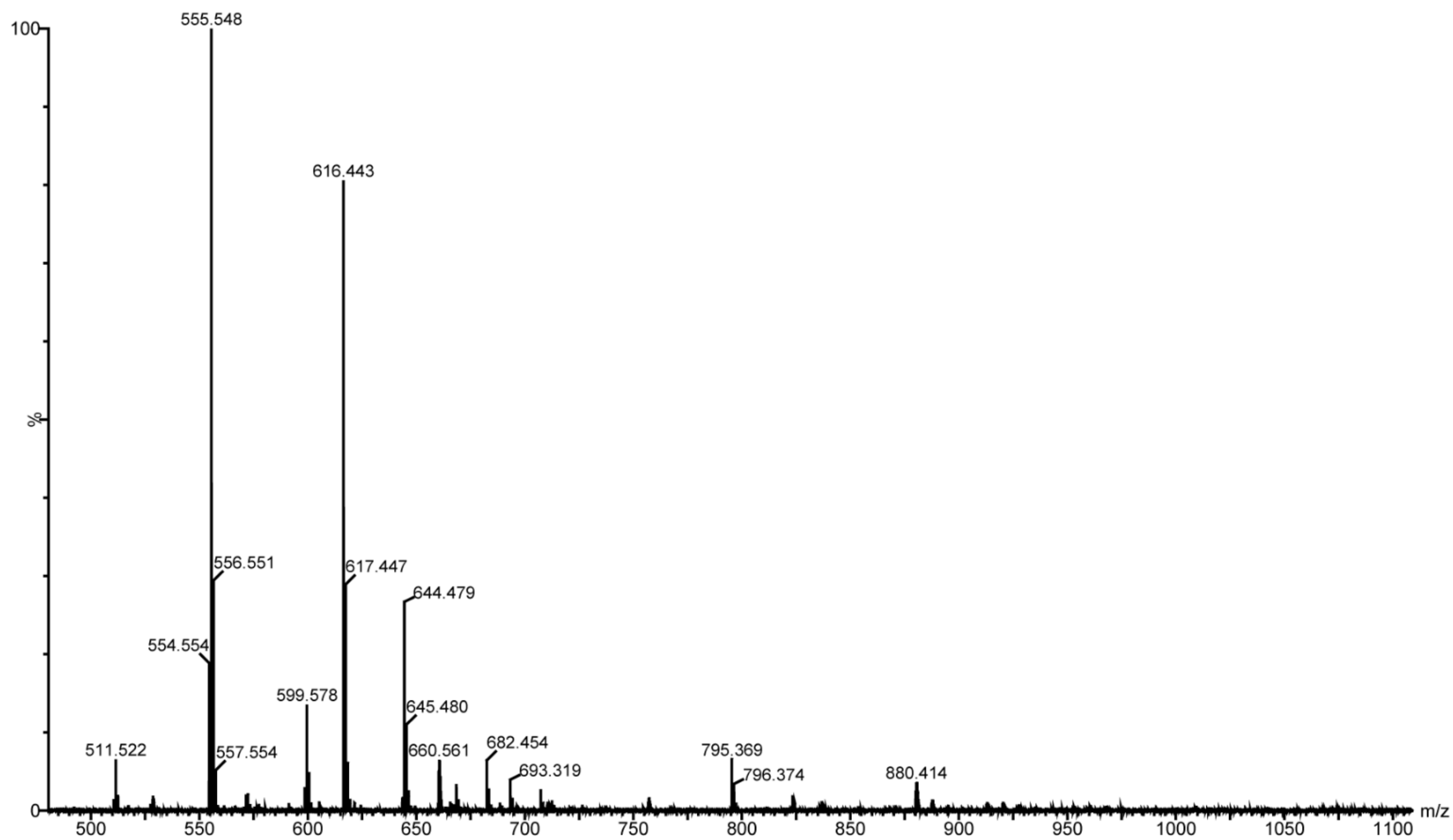

Figure S16 TOF mass spectrometric analysis of the molecular masses of ziconotide degradation products.

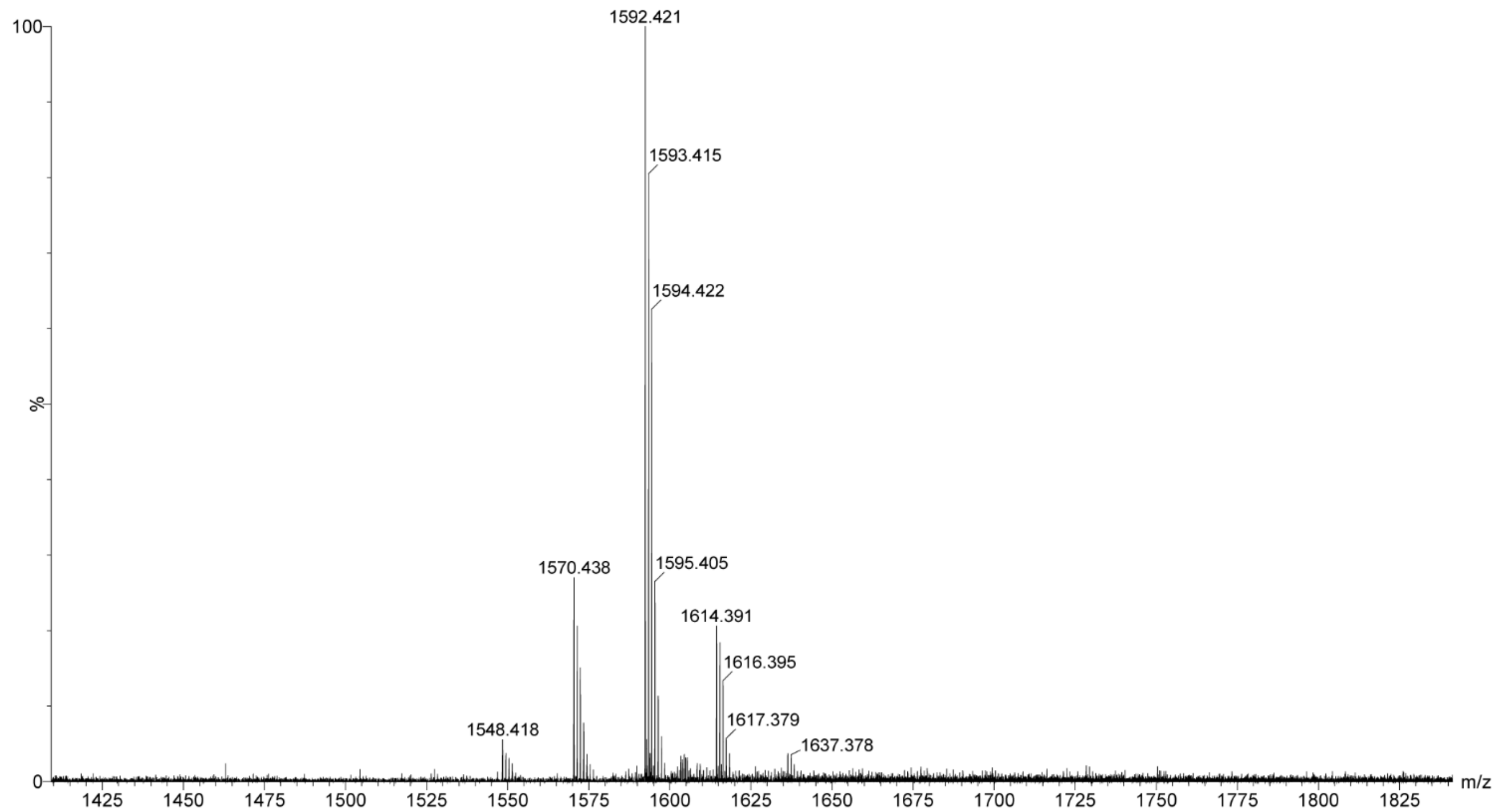

Figure S17 TOF mass spectrometric analysis of the molecular masses of linaclotide degradation products.
